# Supplementary figures and images for: Cell division cycle associated 2 (CDCA2) upregulation promotes the progression of hepatocellular carcinoma in a p53-dependant manner
Source: PeerJ. 2022 Jun 6;10:e13535. doi: 10.7717/peerj.13535 (PMC9179591; doi:10.7717/peerj.13535)

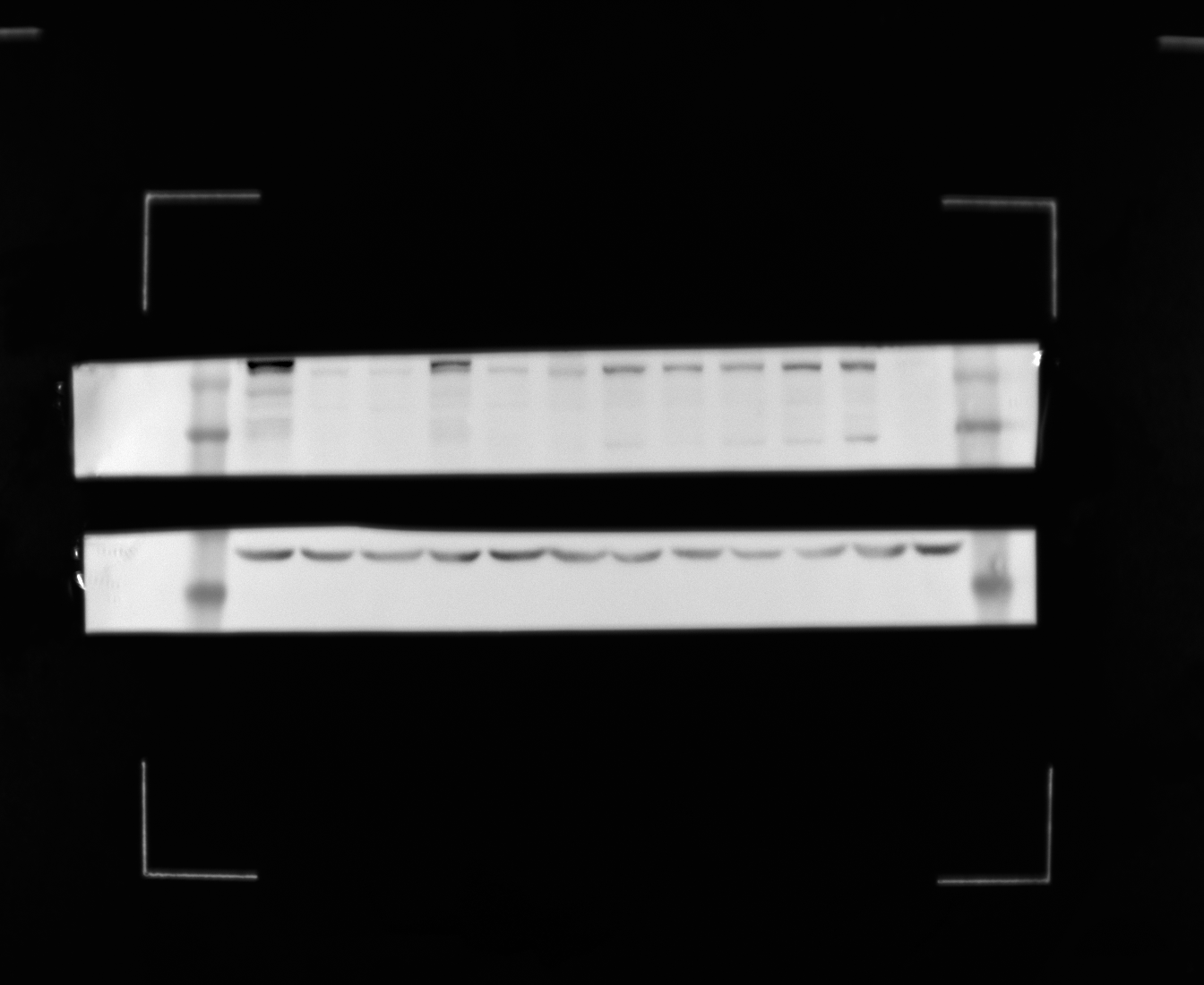

Supplement: Supplemental Information 1 [file peerj-10-13535-s001.zip › Original WB pictures/Fig.1I-CDCA2+Tubulin.tif]

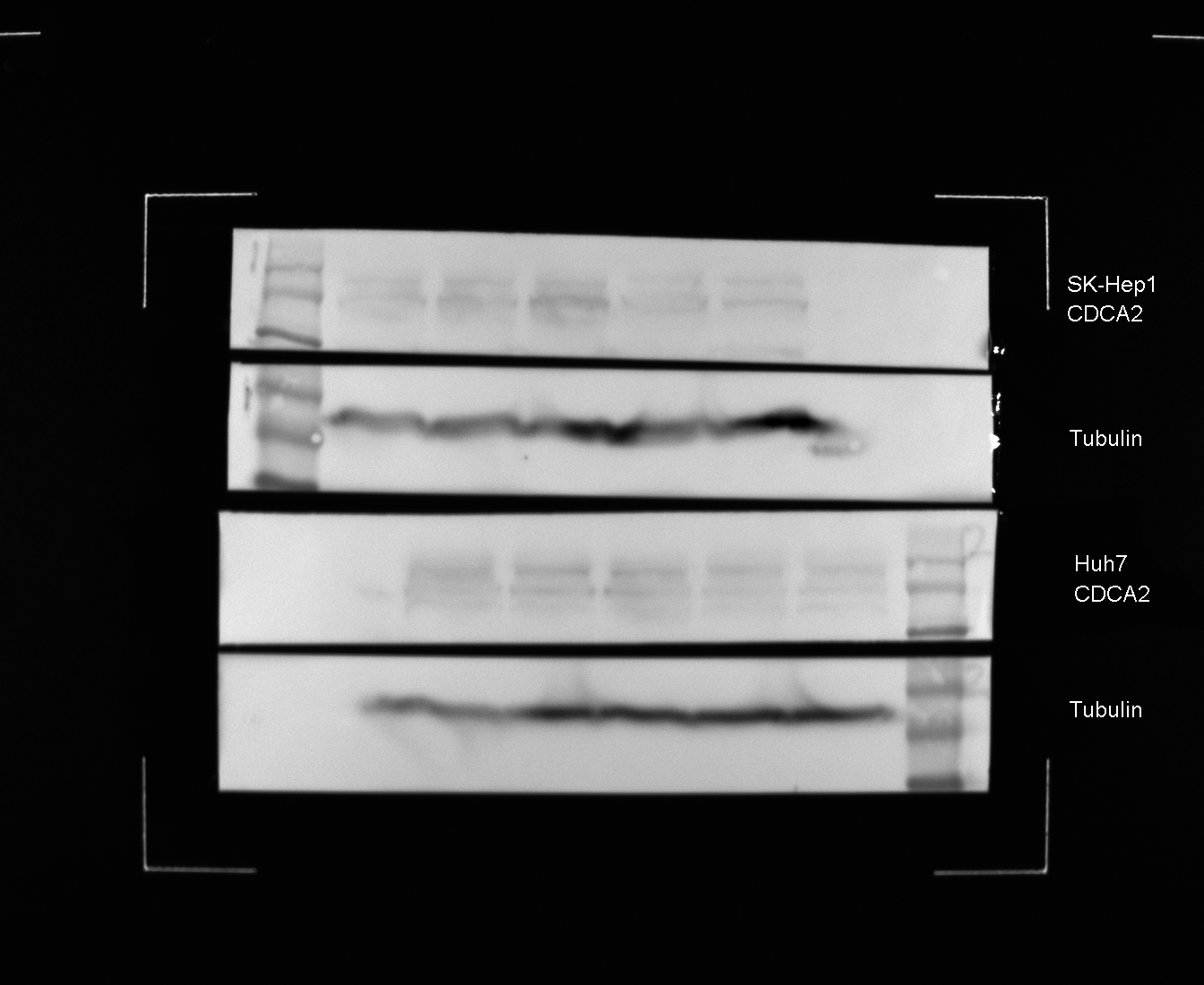

Supplement: Supplemental Information 1 [file peerj-10-13535-s001.zip › Original WB pictures/Fig.1J-CDCA2+Tubulin exposure 1.tif]

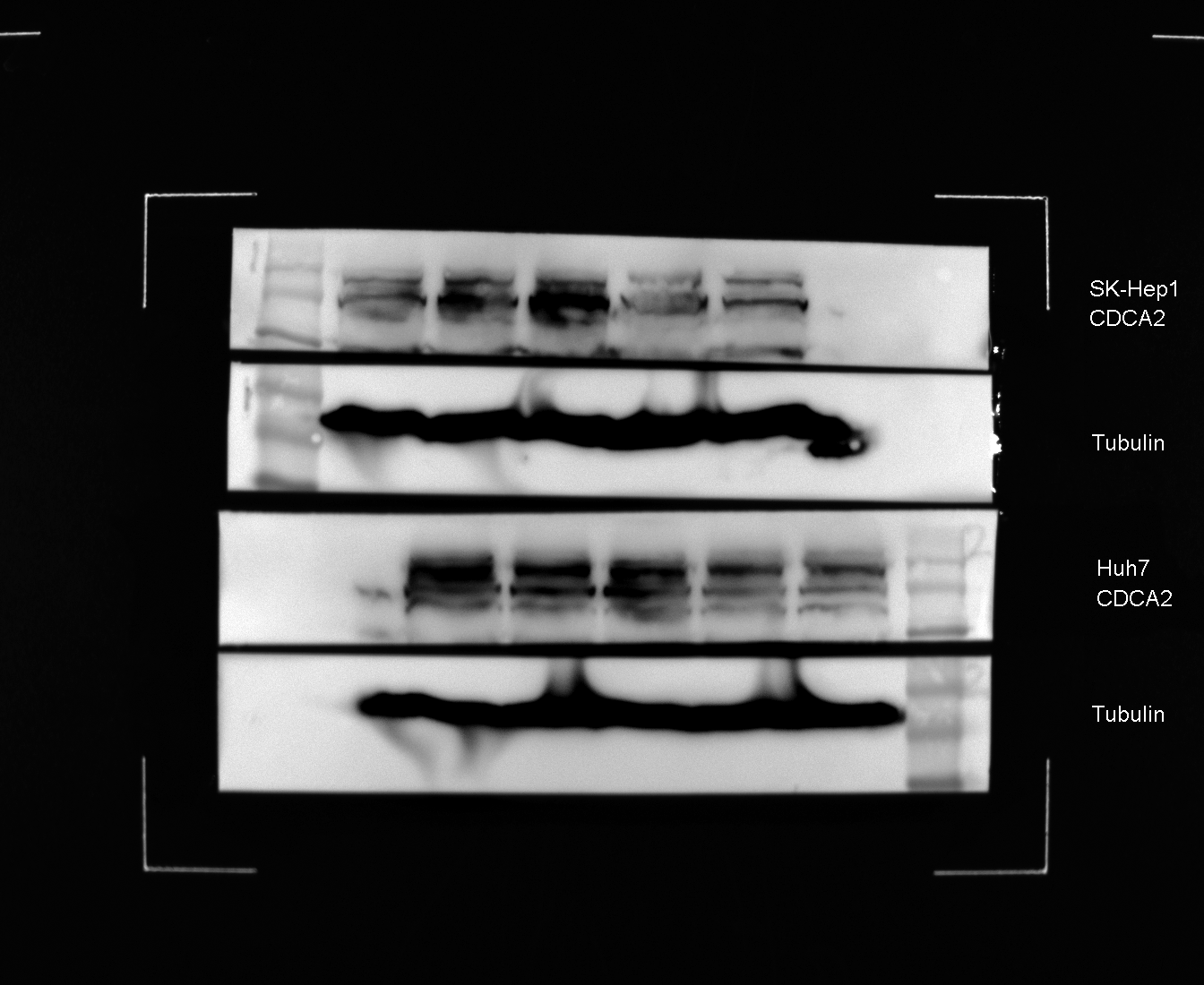

Supplement: Supplemental Information 1 [file peerj-10-13535-s001.zip › Original WB pictures/Fig.1J-CDCA2+Tubulin exposure 2.tif]

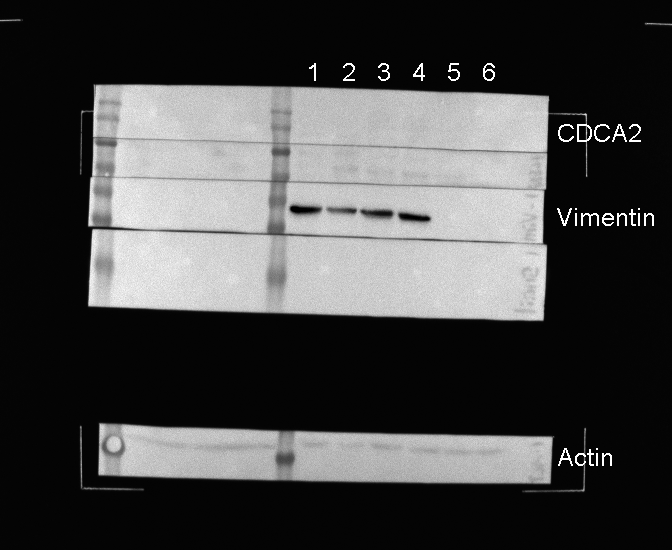

Supplement: Supplemental Information 1 [file peerj-10-13535-s001.zip › Original WB pictures/Fig.2D1-CDCA2+Vimentin+Actin exposure 1.tif]

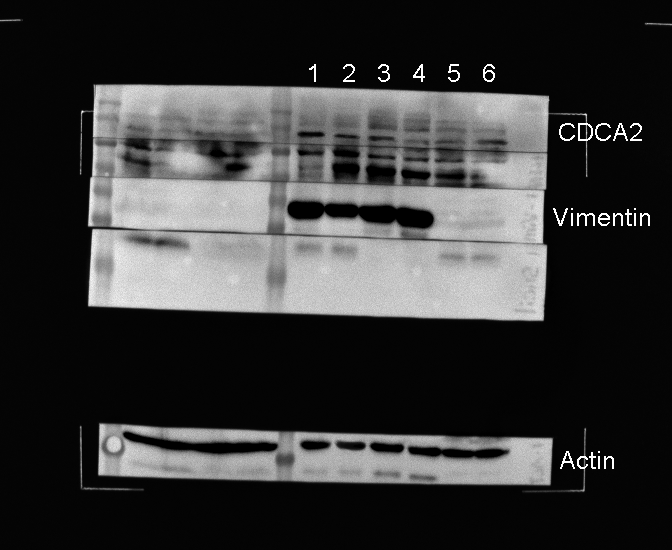

Supplement: Supplemental Information 1 [file peerj-10-13535-s001.zip › Original WB pictures/Fig.2D1-CDCA2+Vimentin+Actin exposure 2.tif]

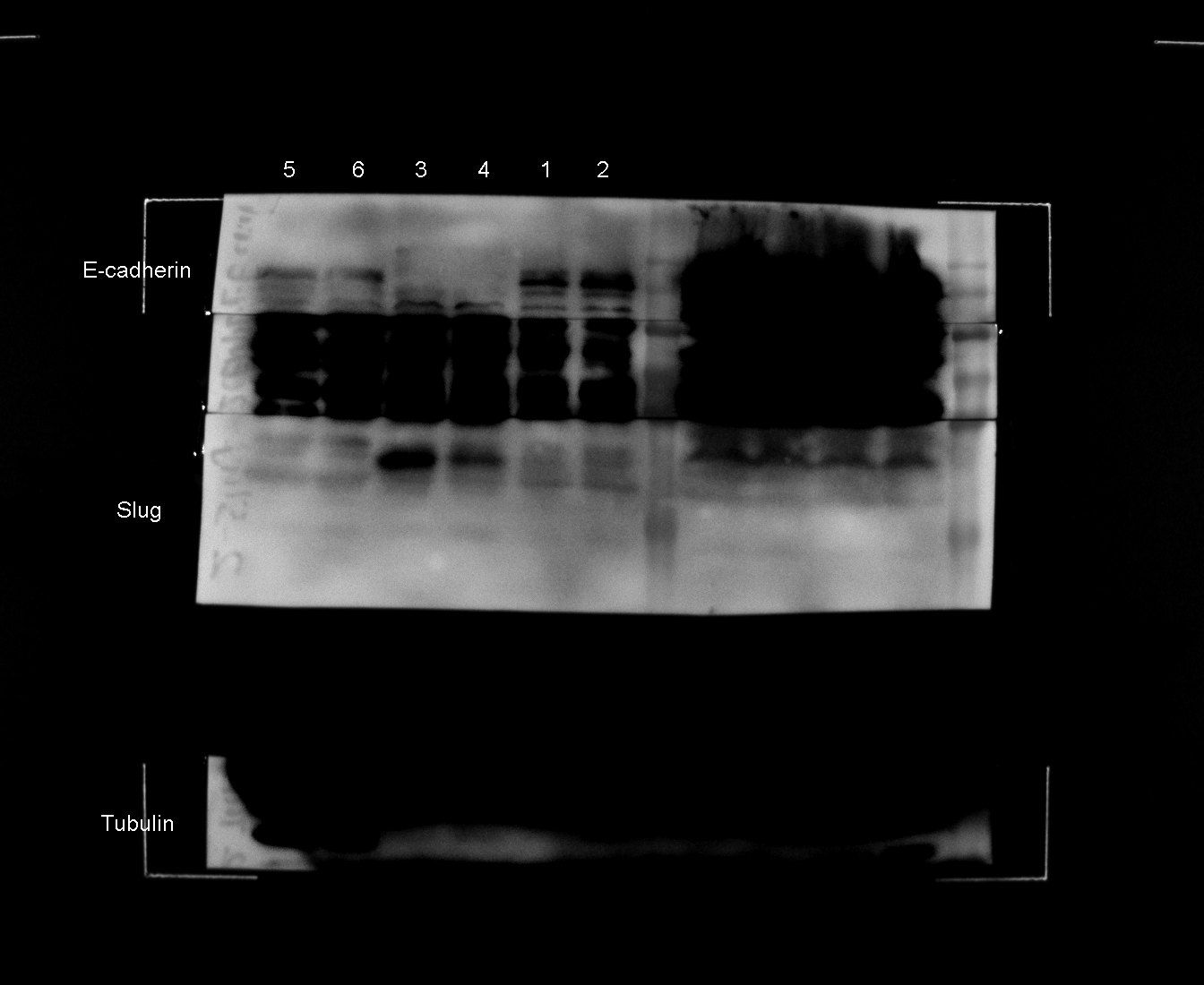

Supplement: Supplemental Information 1 [file peerj-10-13535-s001.zip › Original WB pictures/Fig.2D2-E-Cad+Slug exposure 1.tif]

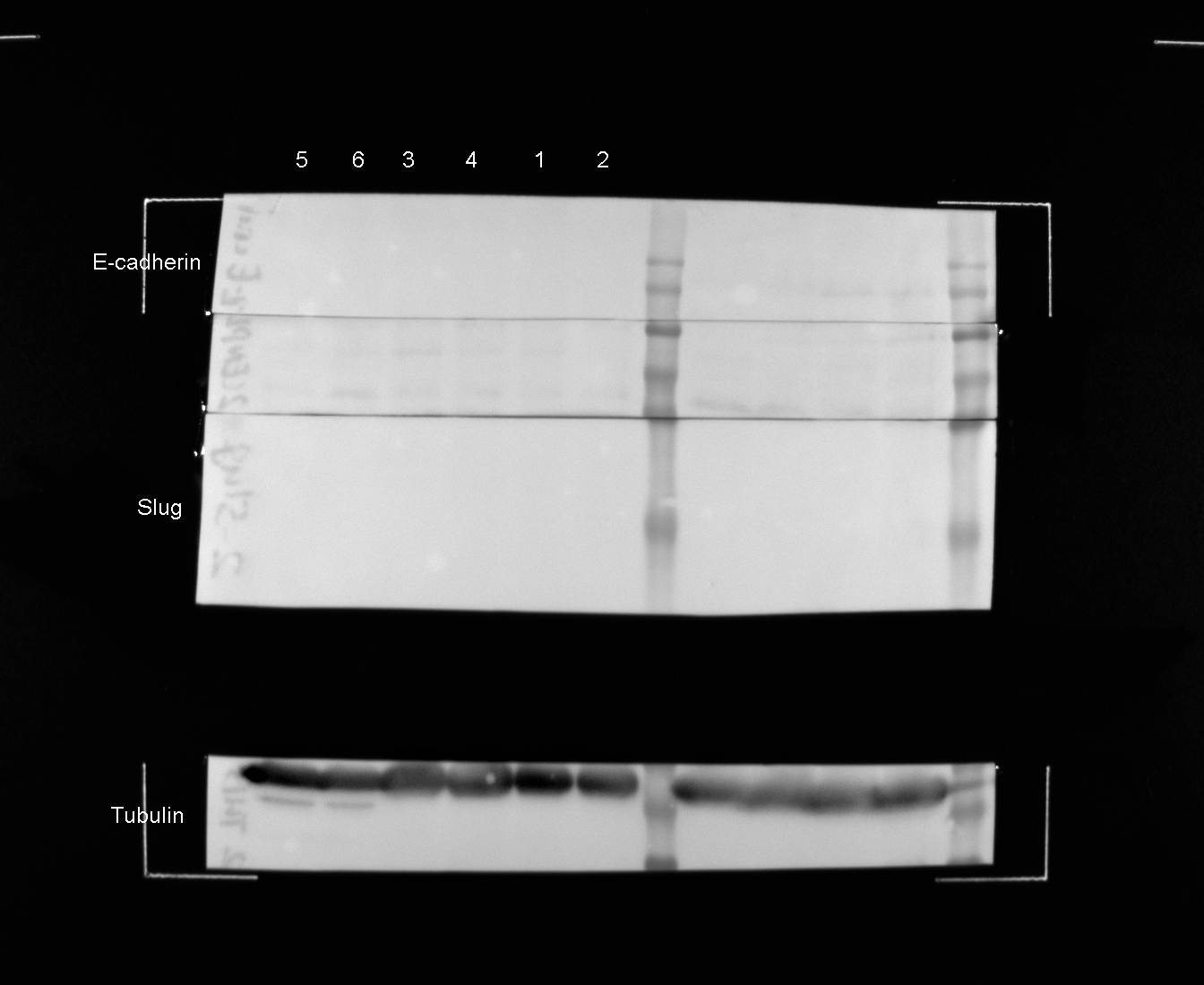

Supplement: Supplemental Information 1 [file peerj-10-13535-s001.zip › Original WB pictures/Fig.2D2-E-Cad+Slug exposure 2.tif]

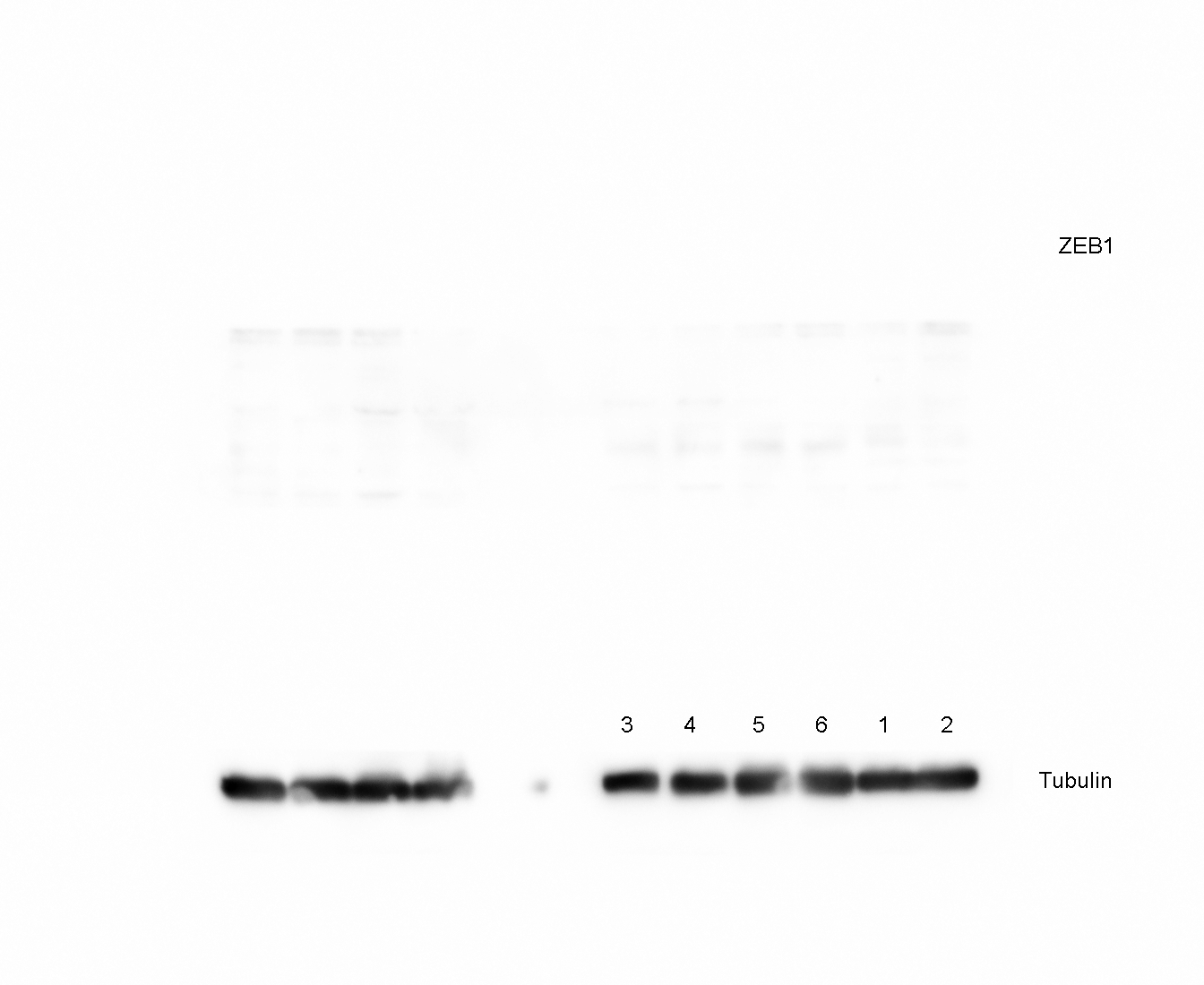

Supplement: Supplemental Information 1 [file peerj-10-13535-s001.zip › Original WB pictures/Fig.2D3-ZEB1+Tubulin exposure 1.tif]

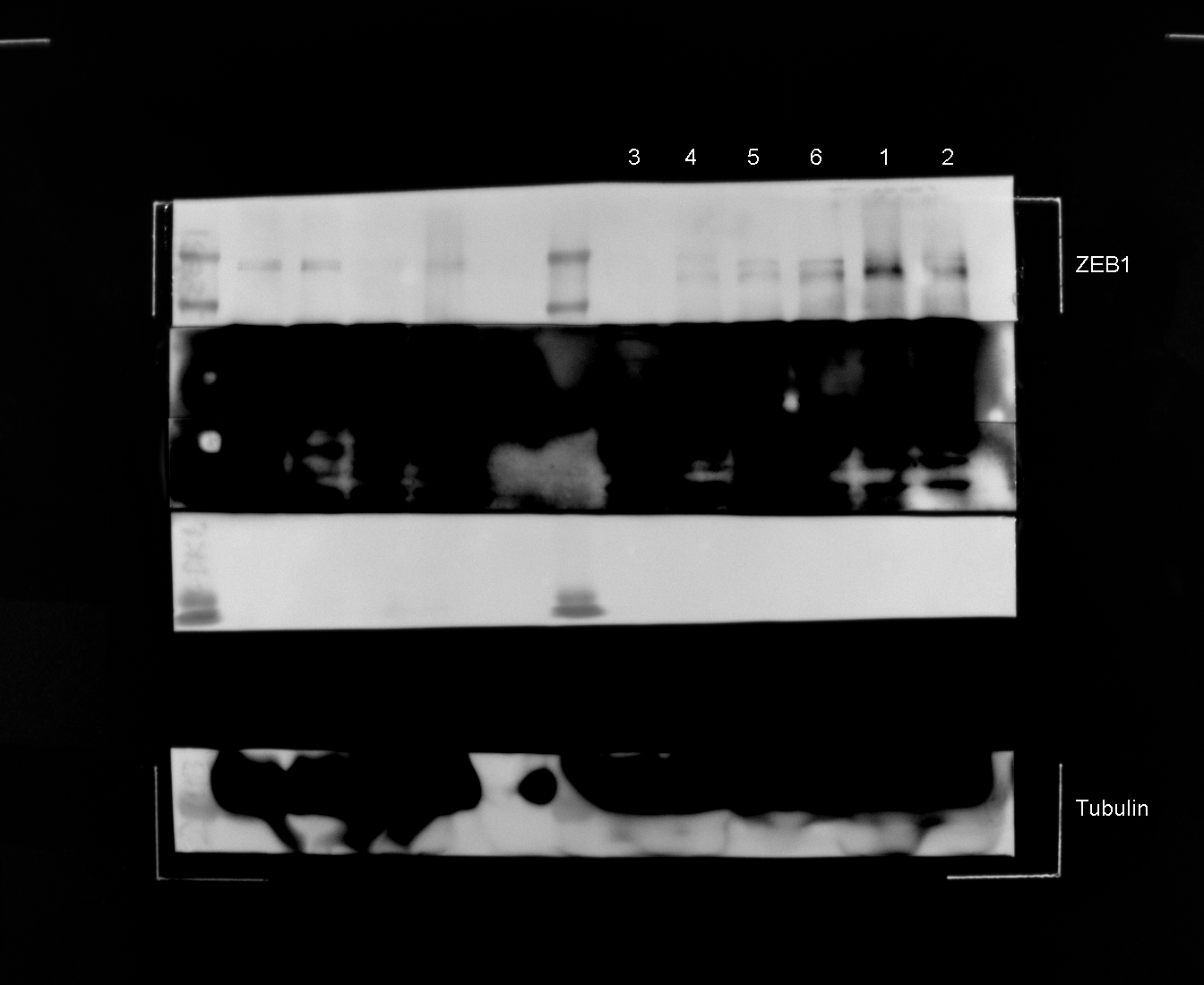

Supplement: Supplemental Information 1 [file peerj-10-13535-s001.zip › Original WB pictures/Fig.2D3-ZEB1+Tubulin exposure 2.tif]

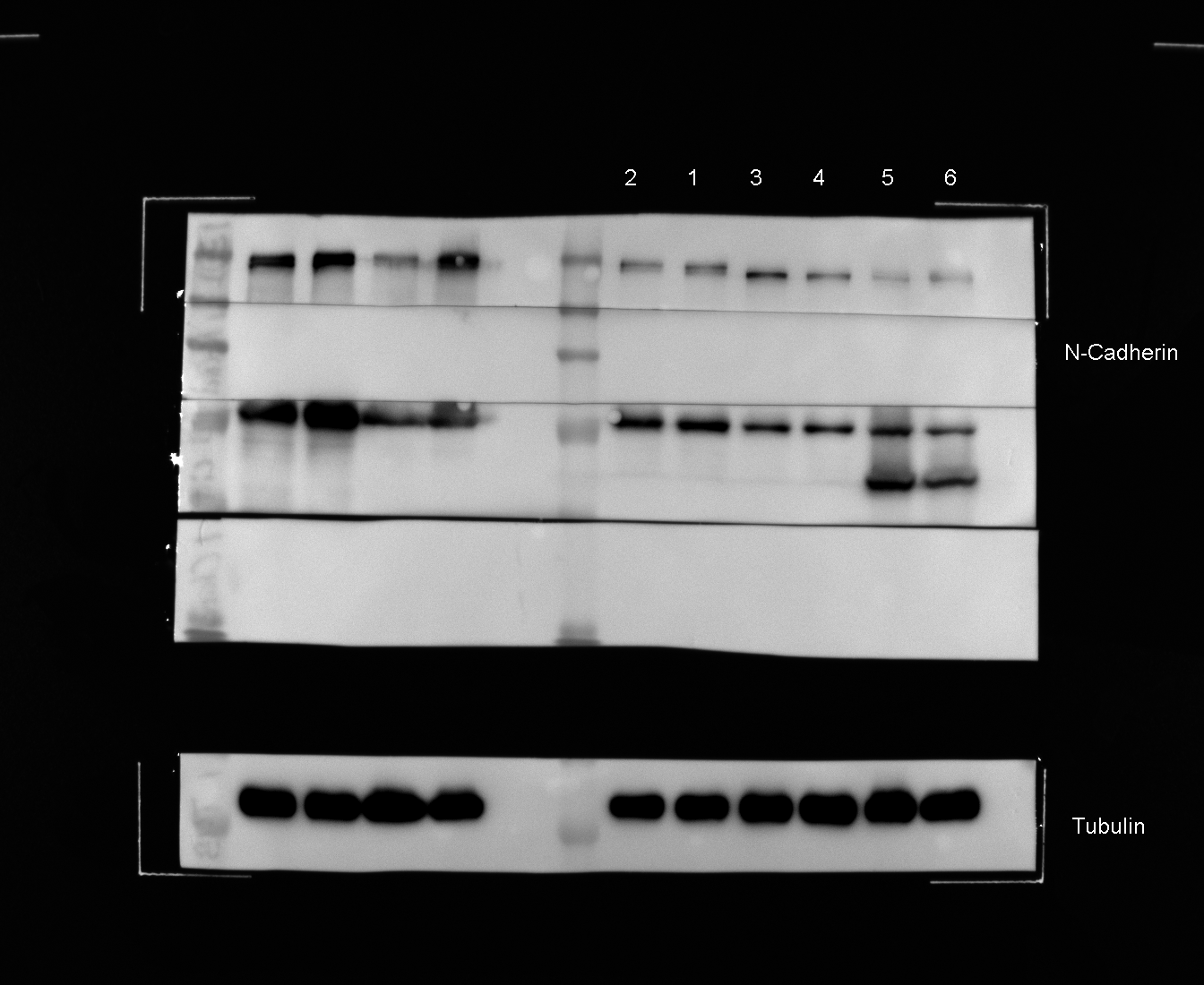

Supplement: Supplemental Information 1 [file peerj-10-13535-s001.zip › Original WB pictures/Fig.2D4-N-cadherin+Tubulin exposure 1.tif]

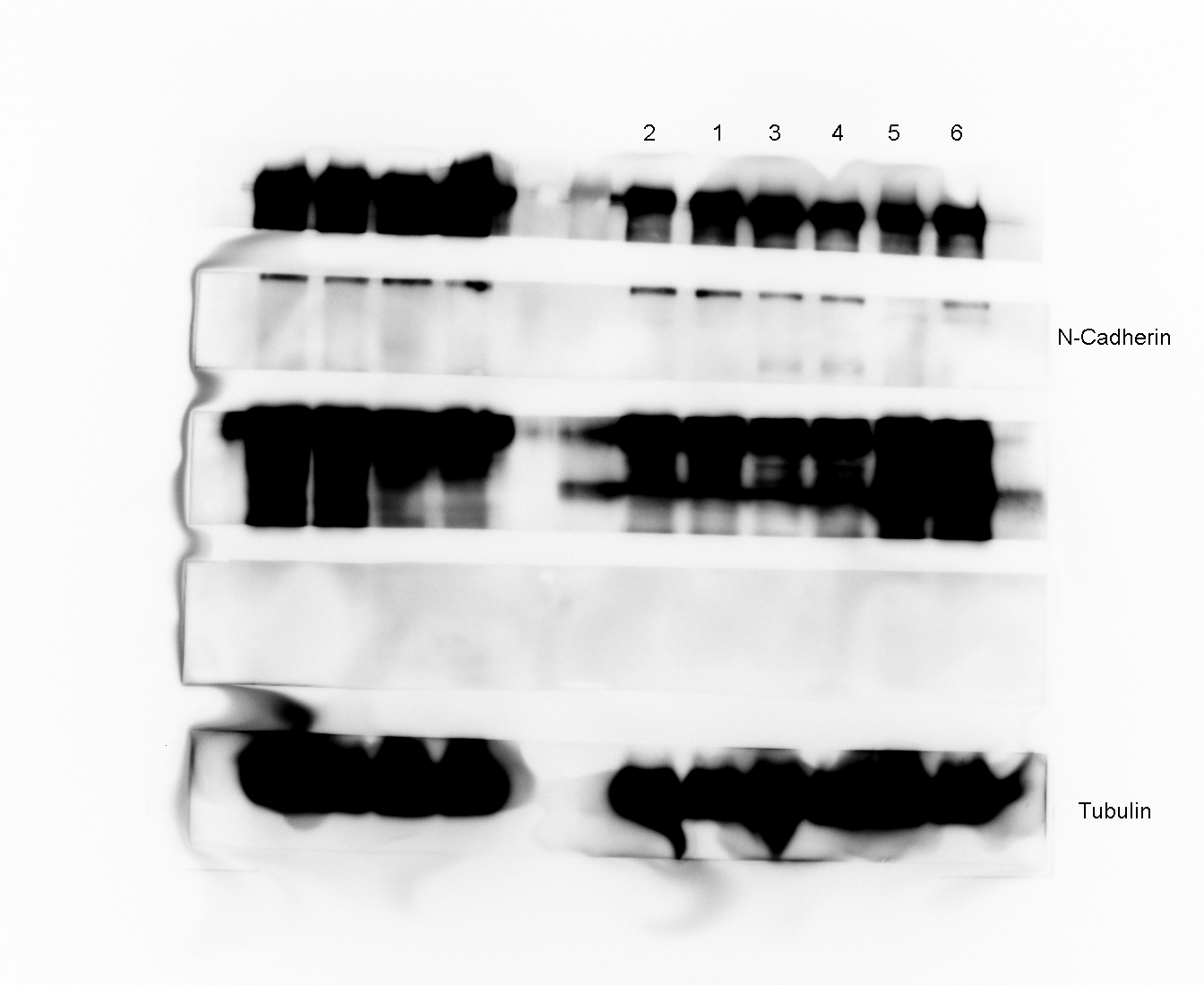

Supplement: Supplemental Information 1 [file peerj-10-13535-s001.zip › Original WB pictures/Fig.2D4-N-cadherin+Tubulin exposure 2-membranes seperated to avoid overflow from other bands.tif]

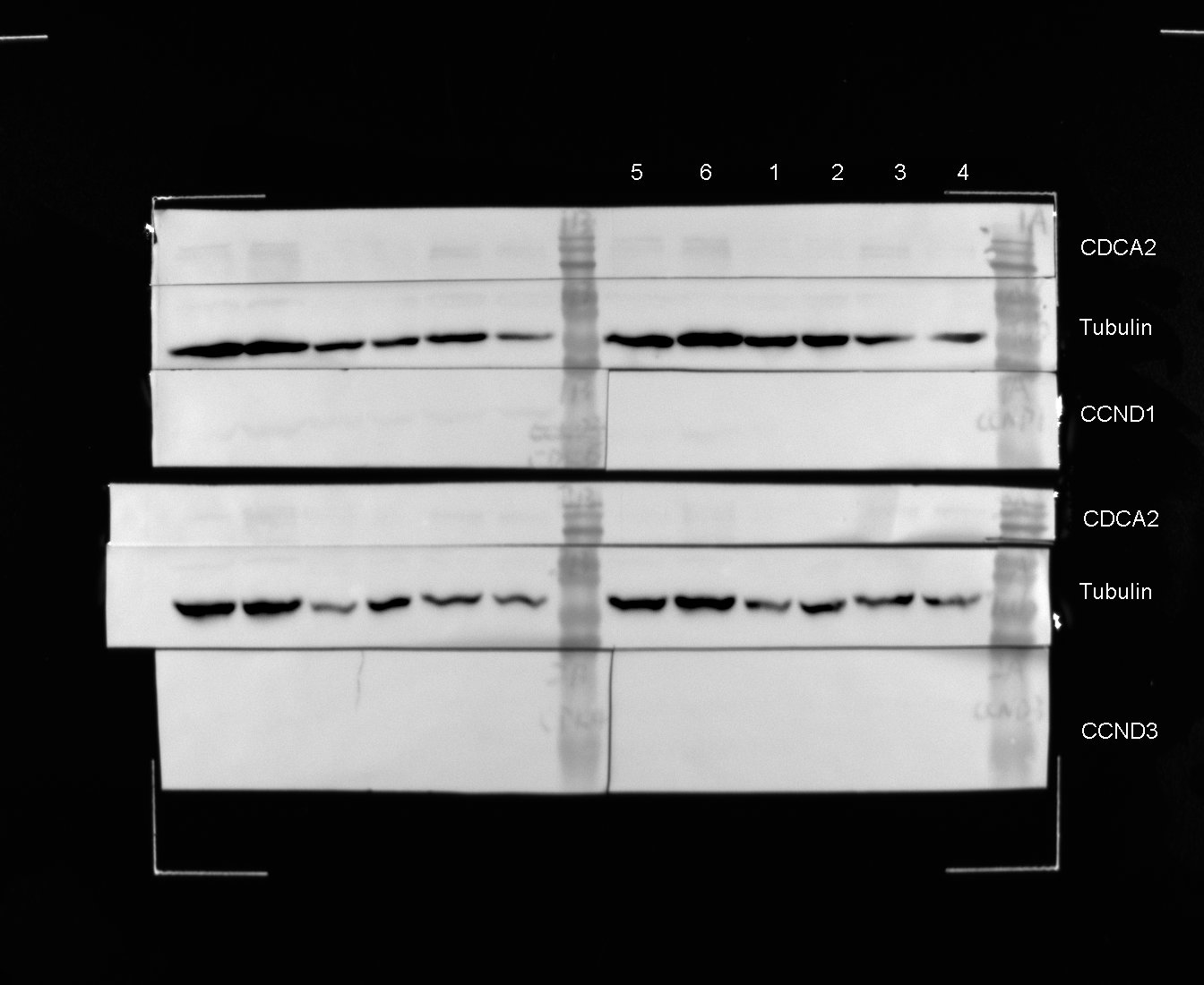

Supplement: Supplemental Information 1 [file peerj-10-13535-s001.zip › Original WB pictures/Fig.2F1-CCND1+CCND3+Tubulin exposure 1.tif]

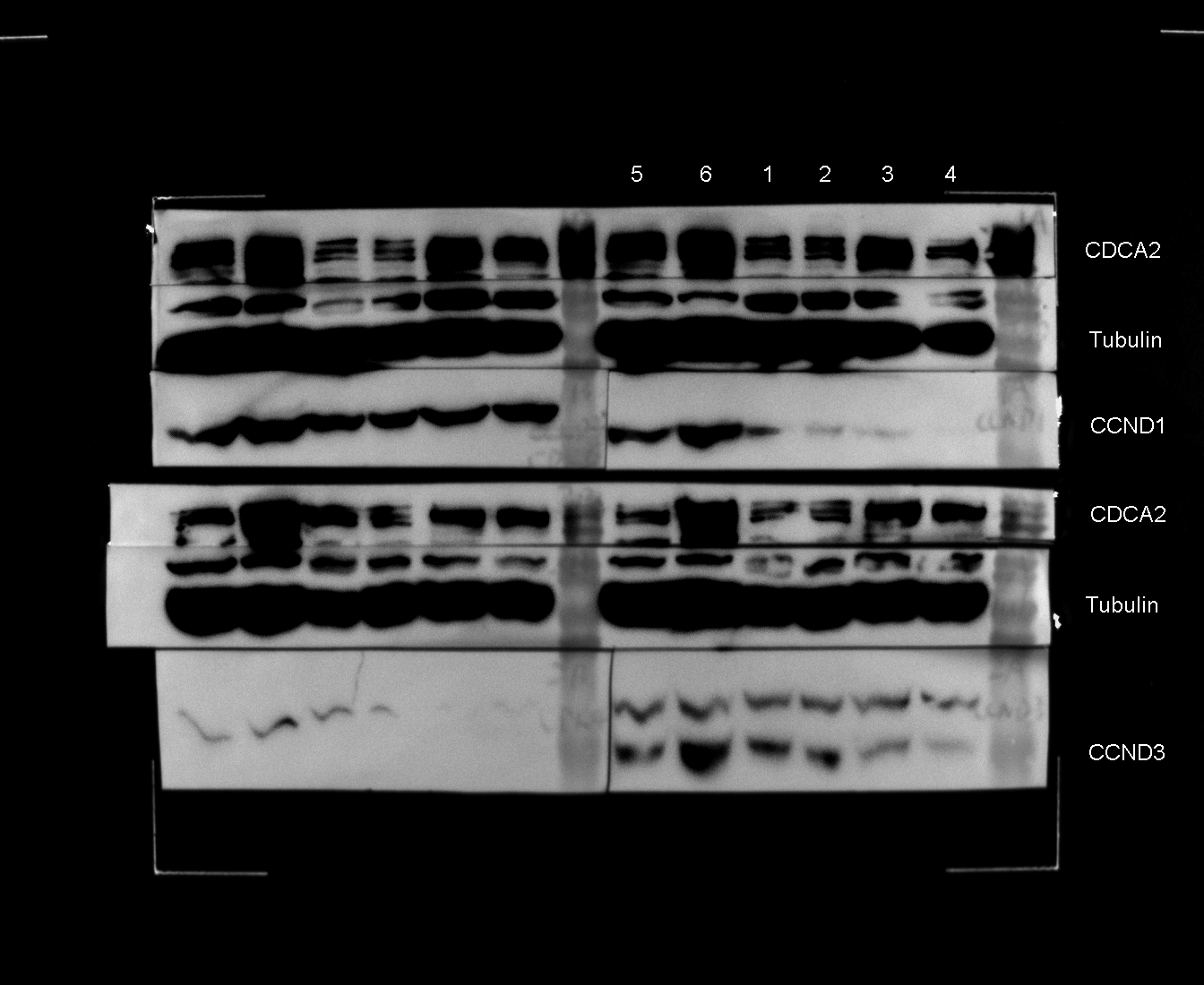

Supplement: Supplemental Information 1 [file peerj-10-13535-s001.zip › Original WB pictures/Fig.2F1-CCND1+CCND3+Tubulin exposure 2.tif]

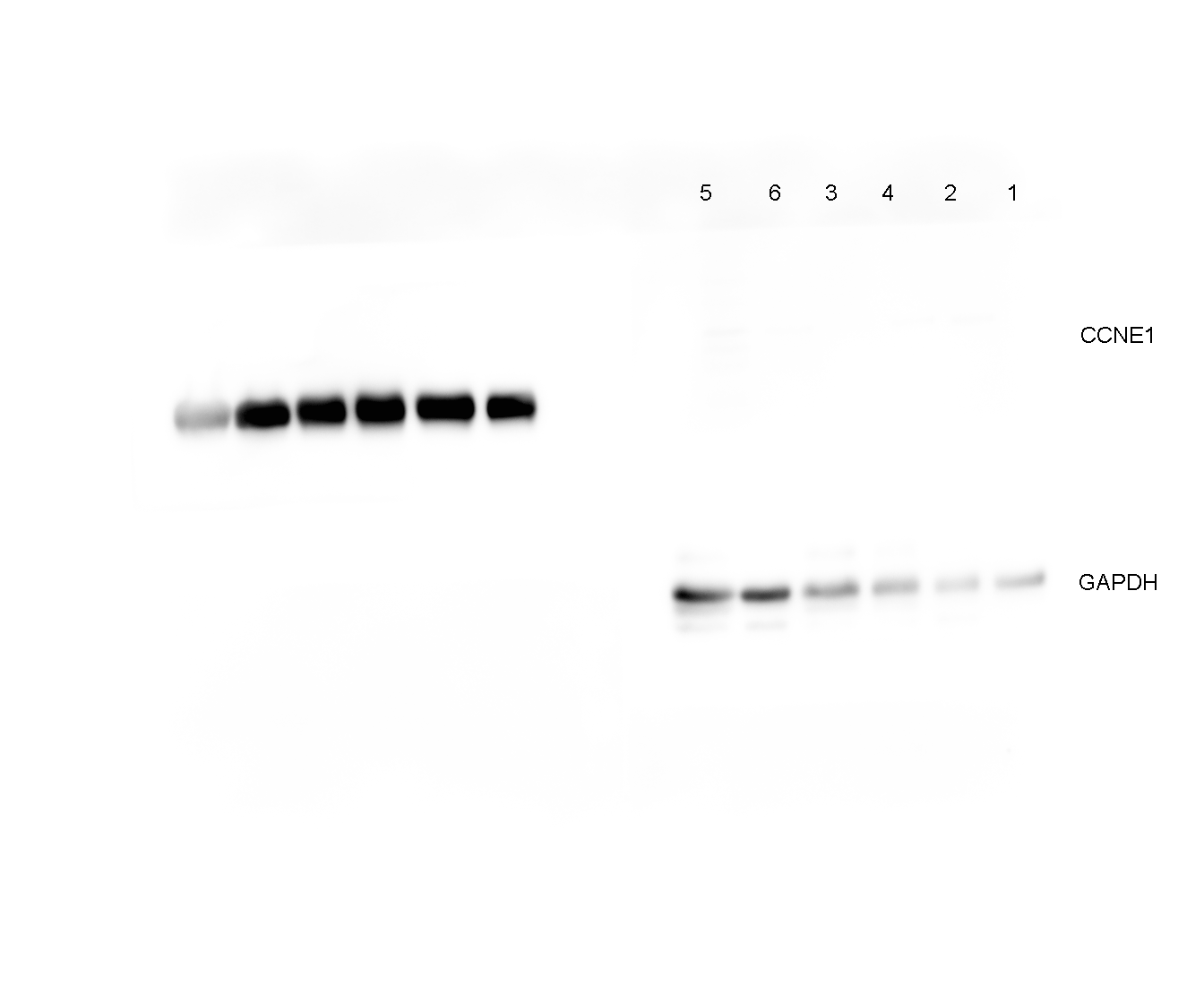

Supplement: Supplemental Information 1 [file peerj-10-13535-s001.zip › Original WB pictures/Fig.2F2-CCNE1+GAPDH exposure 1.tif]

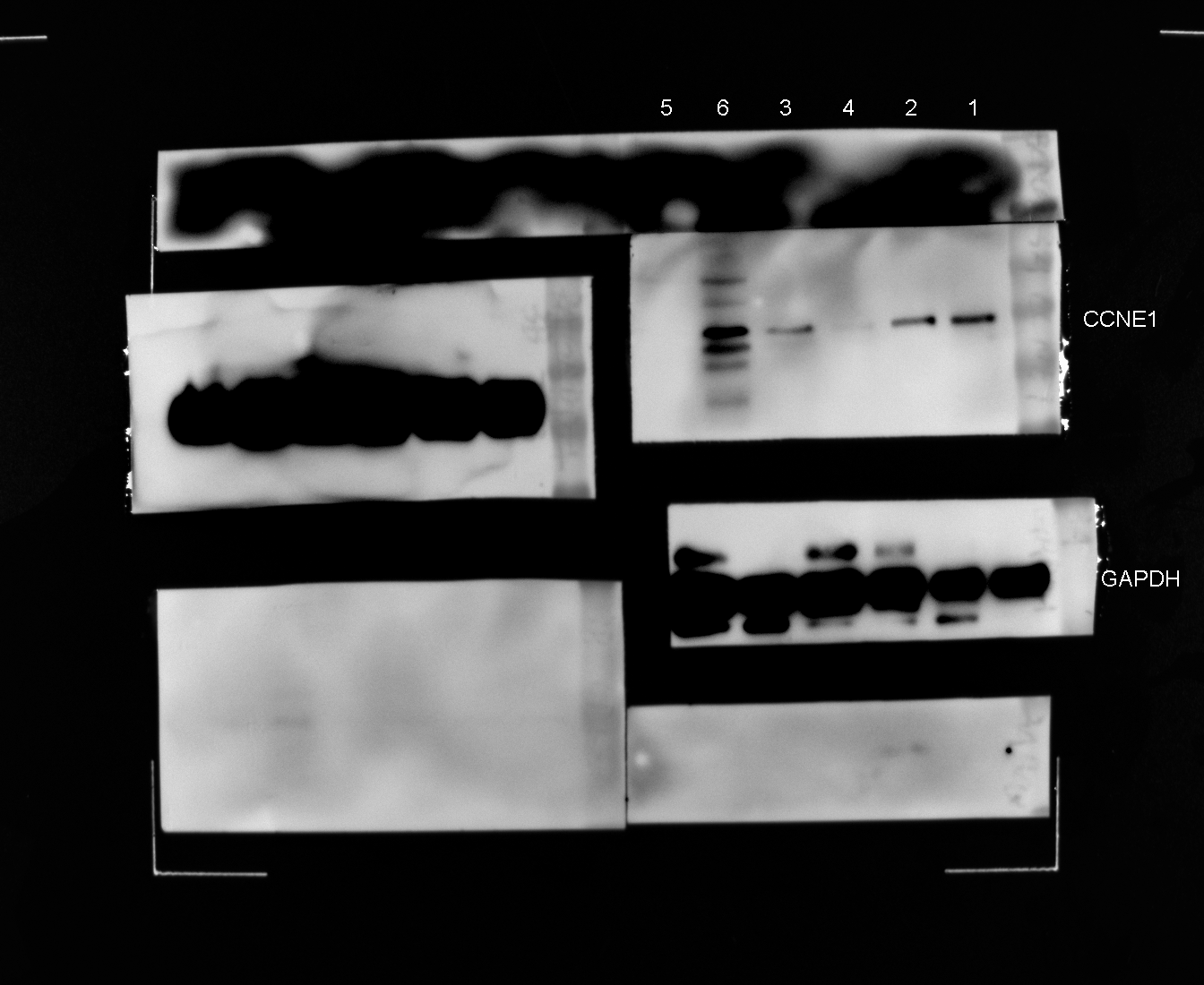

Supplement: Supplemental Information 1 [file peerj-10-13535-s001.zip › Original WB pictures/Fig.2F2-CCNE1+GAPDH exposure 2.tif]

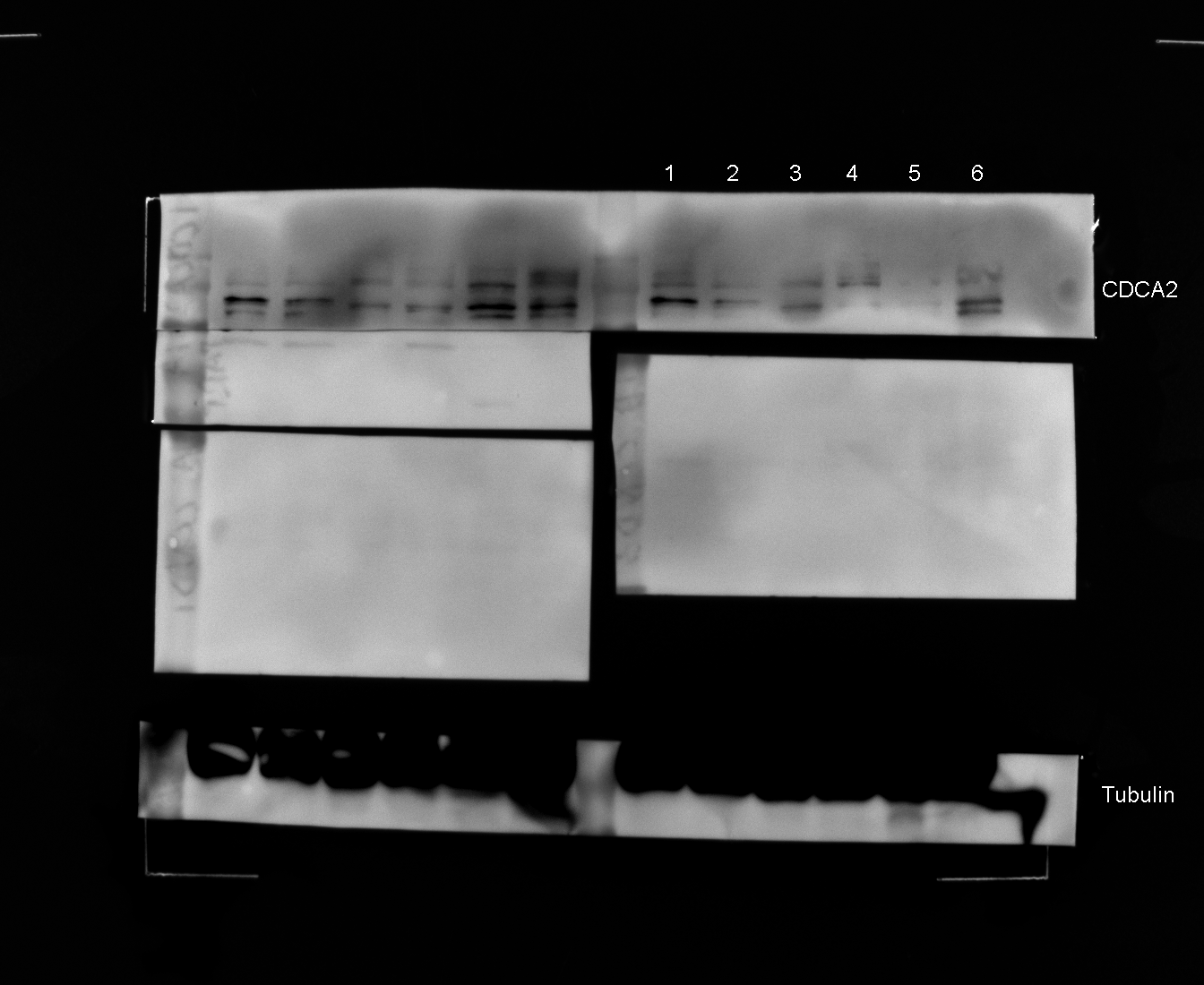

Supplement: Supplemental Information 1 [file peerj-10-13535-s001.zip › Original WB pictures/Fig.2F3-CDCA2.tif]

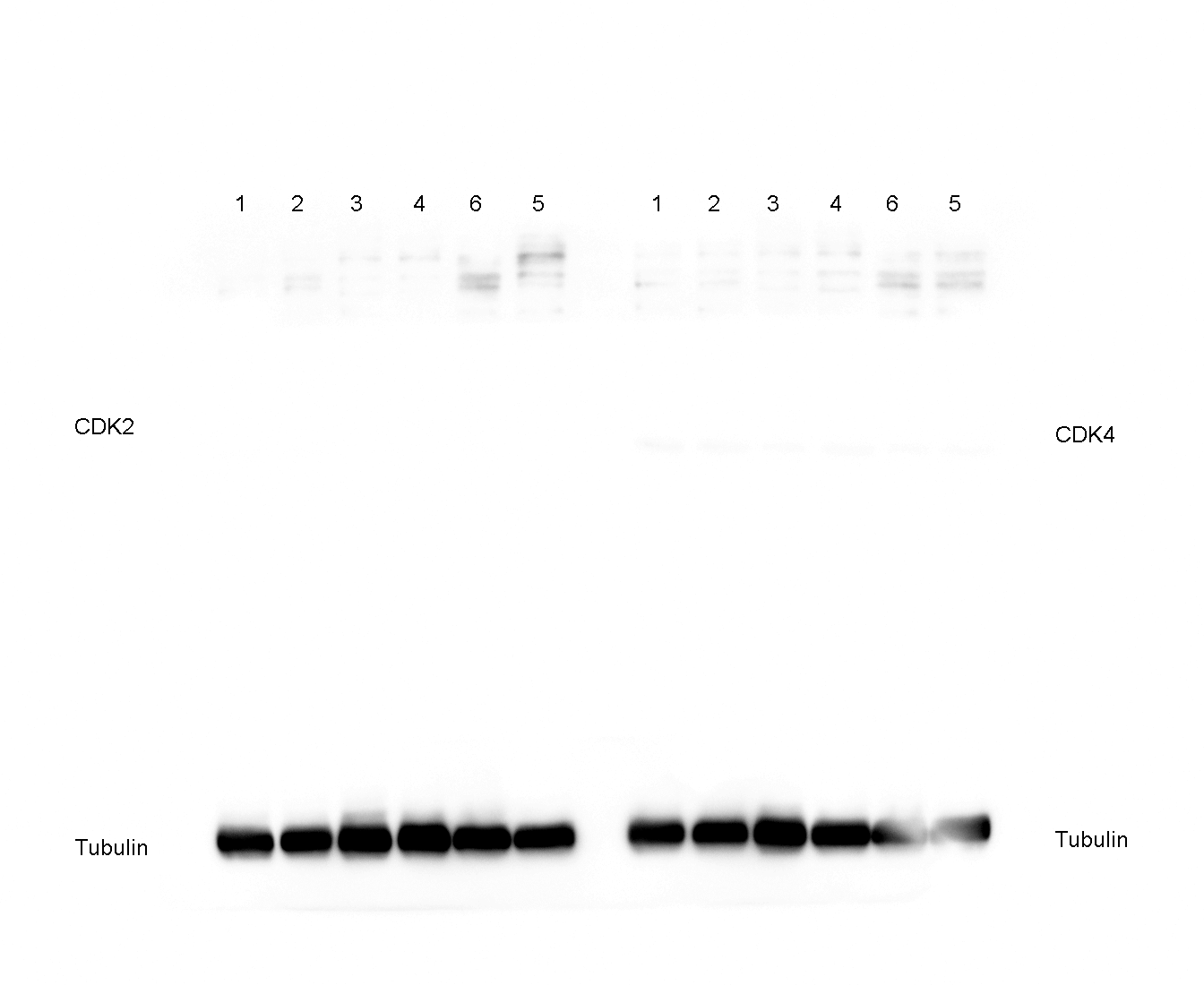

Supplement: Supplemental Information 1 [file peerj-10-13535-s001.zip › Original WB pictures/Fig.2F4-Cdk2+Cdk4+Tubulin exposure 1.tif]

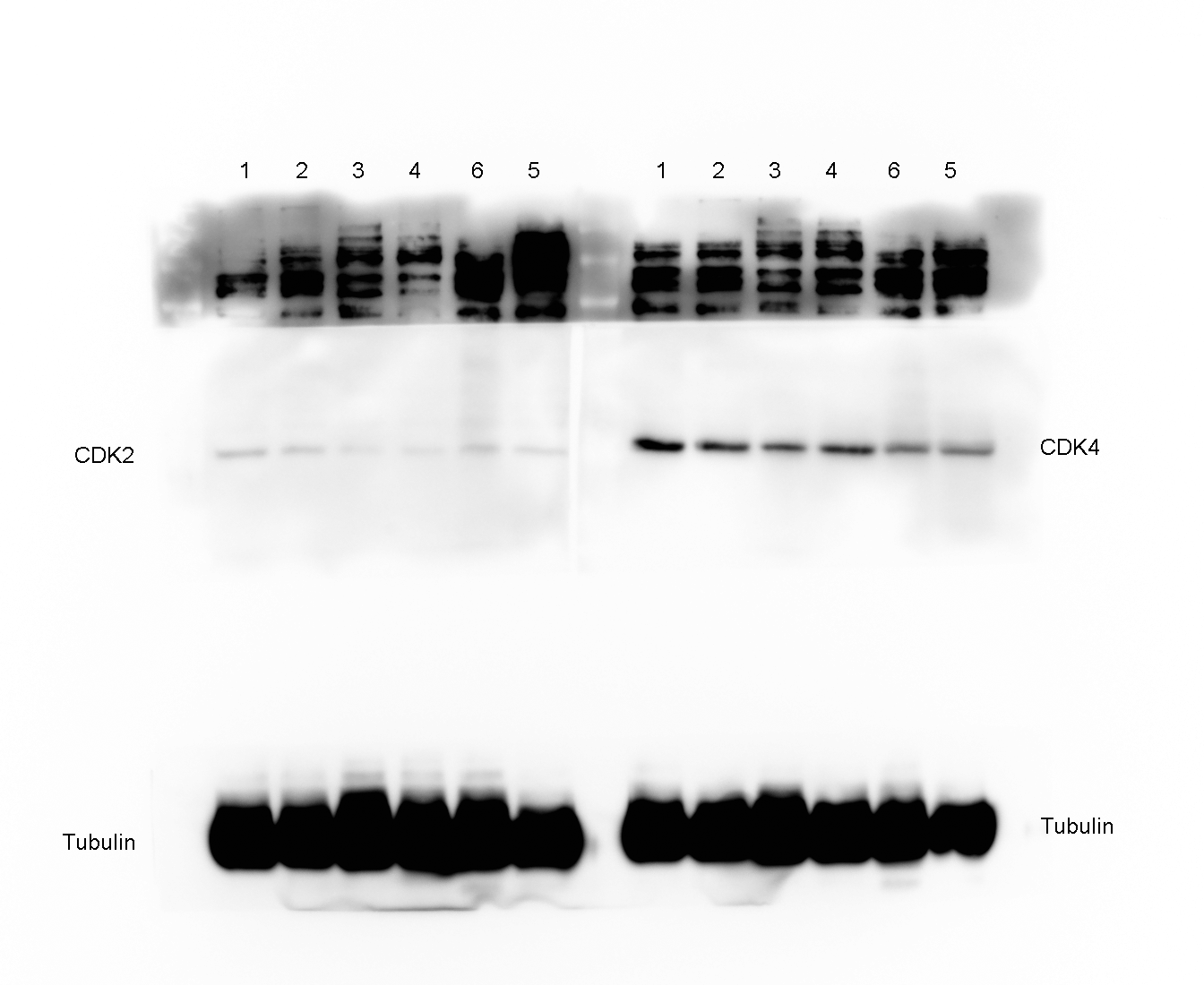

Supplement: Supplemental Information 1 [file peerj-10-13535-s001.zip › Original WB pictures/Fig.2F4-Cdk2+Cdk4+Tubulin exposure 2.tif]

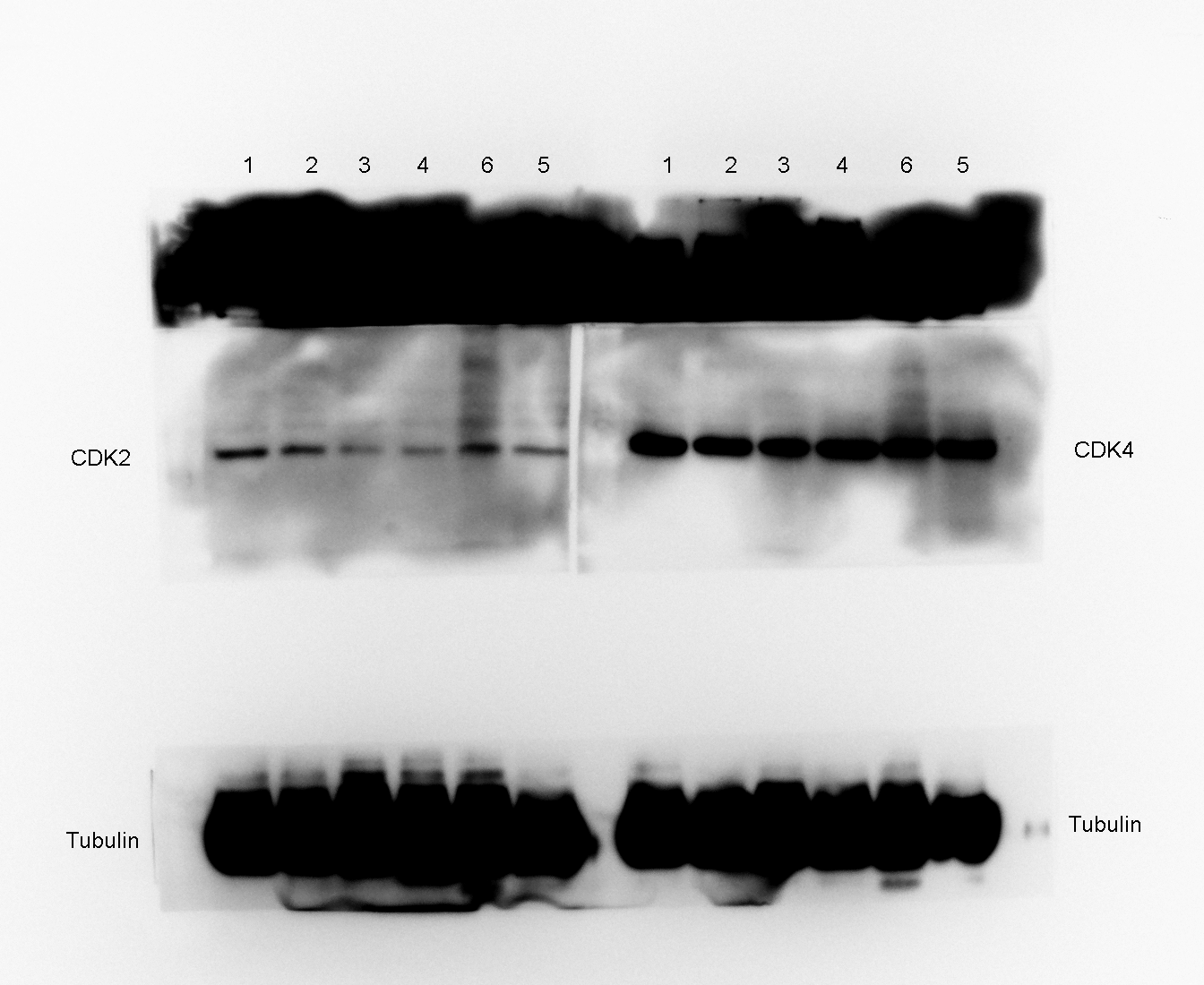

Supplement: Supplemental Information 1 [file peerj-10-13535-s001.zip › Original WB pictures/Fig.2F4-Cdk2+Cdk4+Tubulin exposure 3.tif]

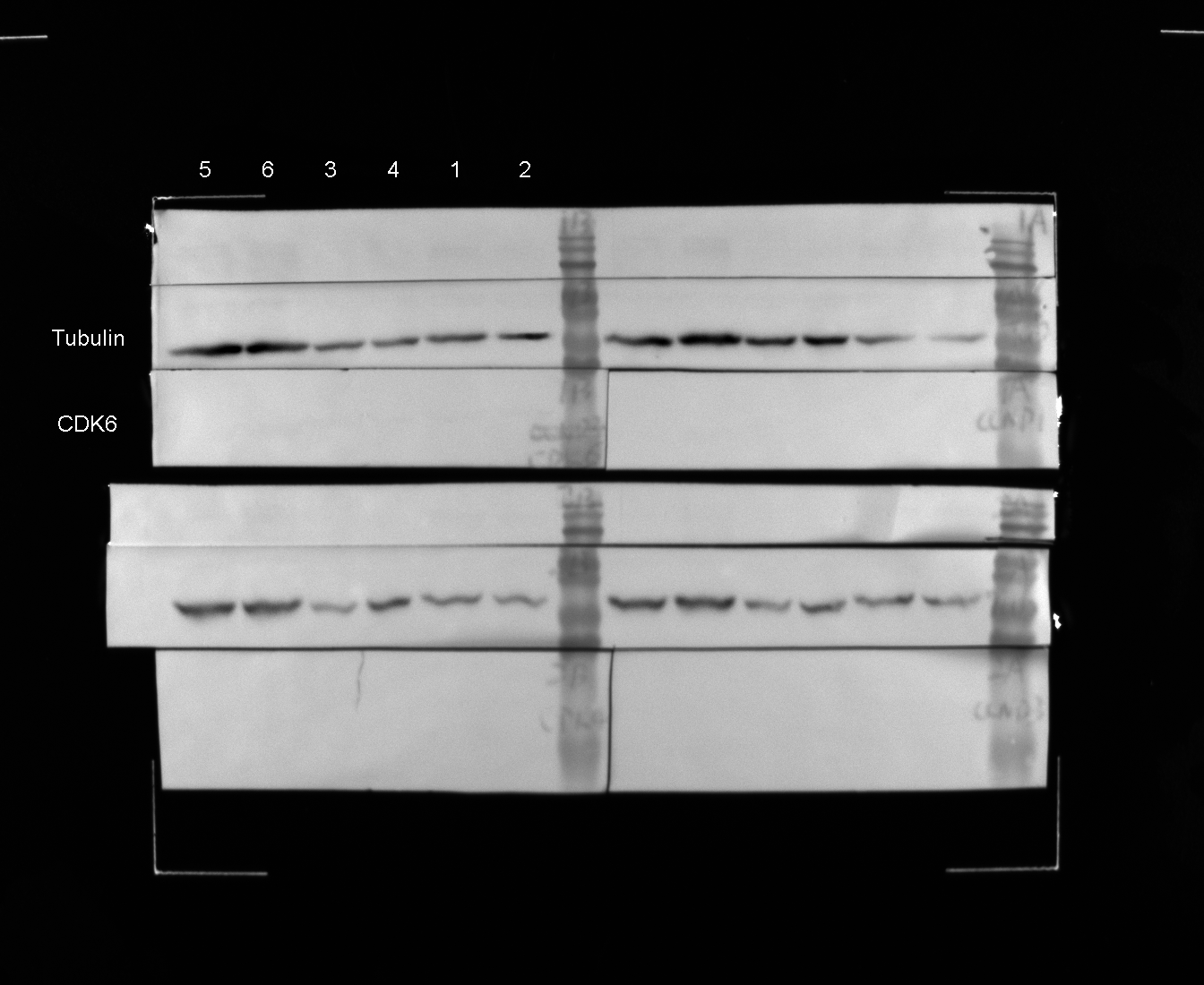

Supplement: Supplemental Information 1 [file peerj-10-13535-s001.zip › Original WB pictures/Fig.2F5-Cdk6+Tubulin exposure 1.tif]

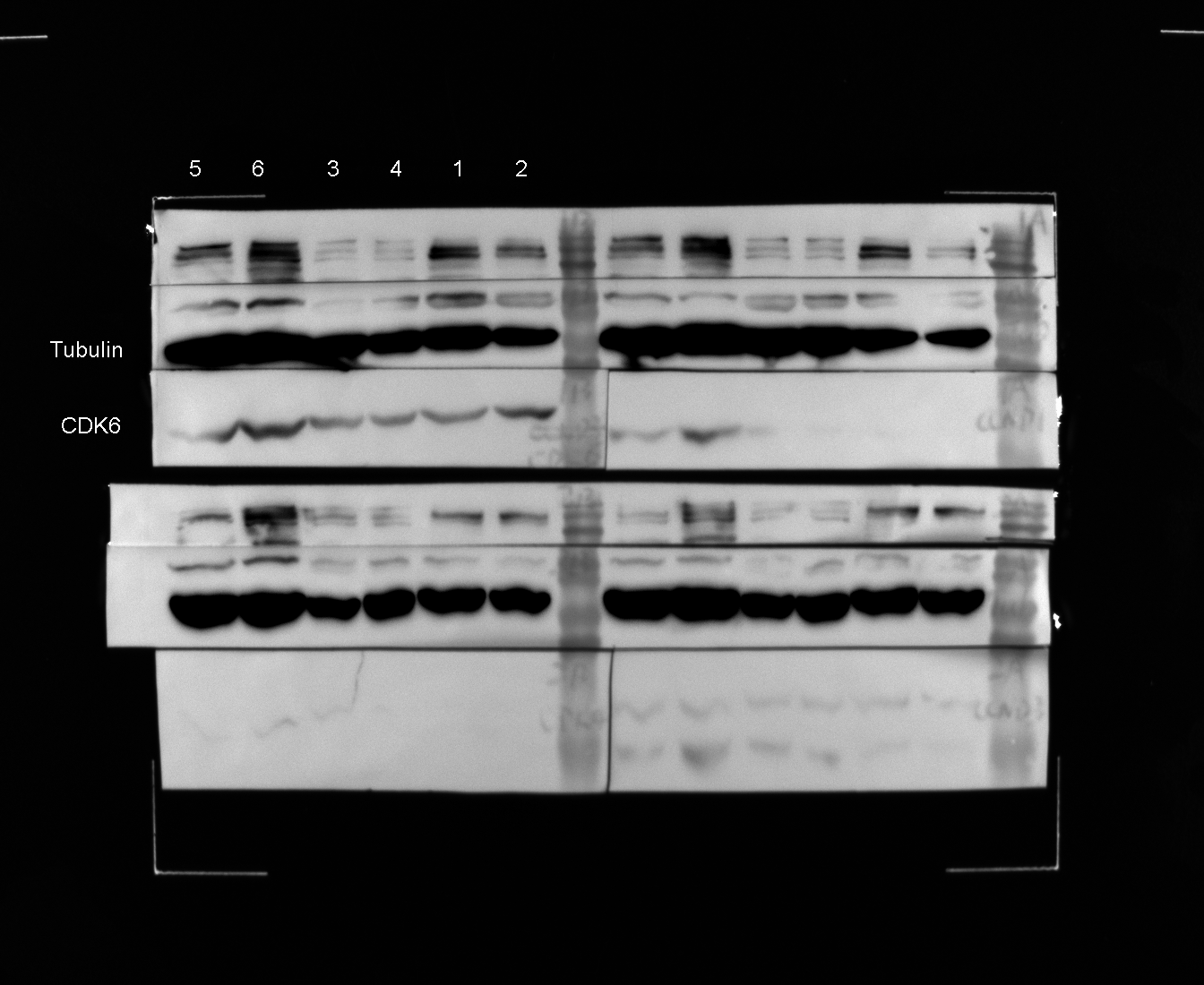

Supplement: Supplemental Information 1 [file peerj-10-13535-s001.zip › Original WB pictures/Fig.2F5-Cdk6+Tubulin exposure 2.tif]

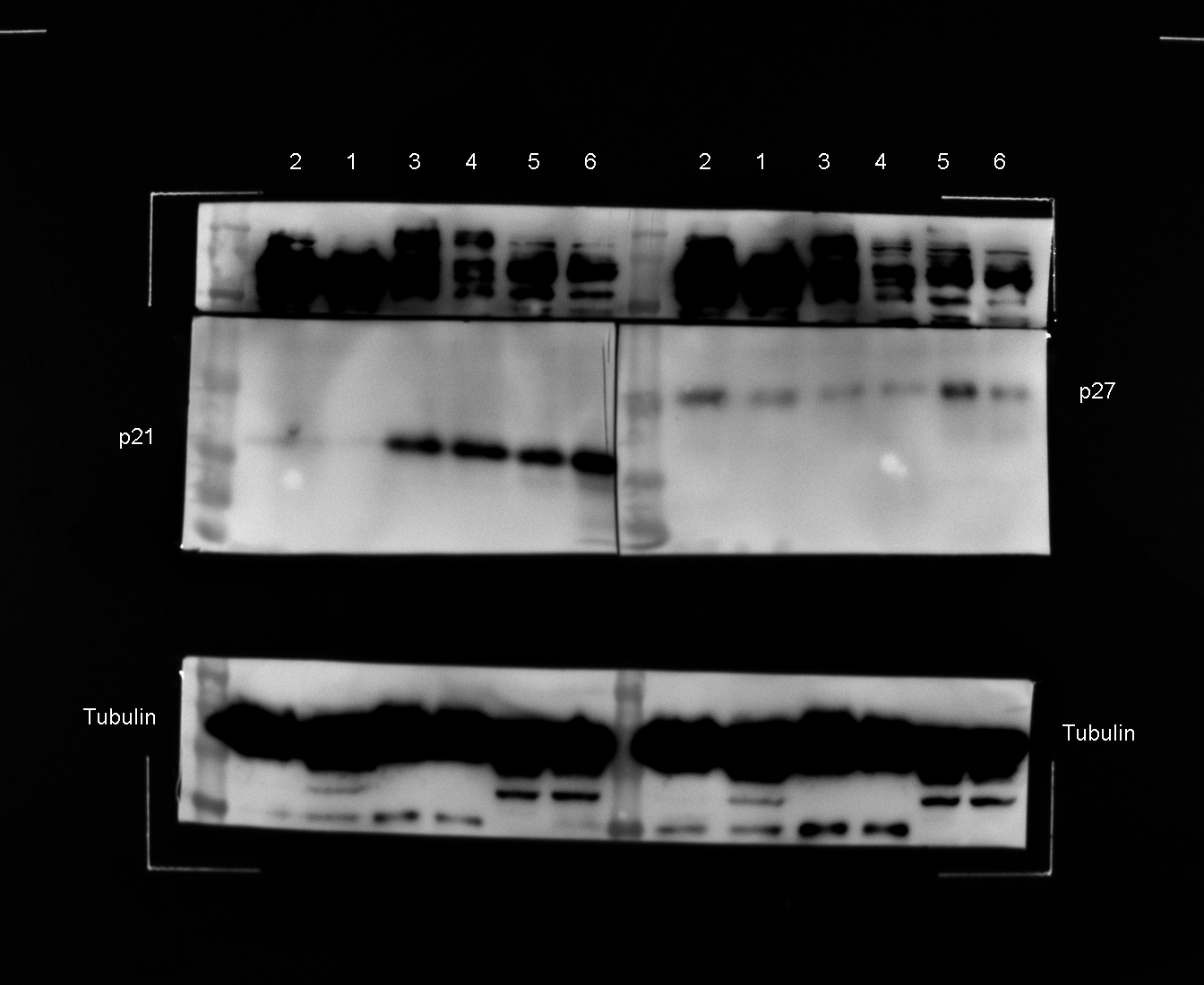

Supplement: Supplemental Information 1 [file peerj-10-13535-s001.zip › Original WB pictures/Fig.2F6-p21+P27.tif]

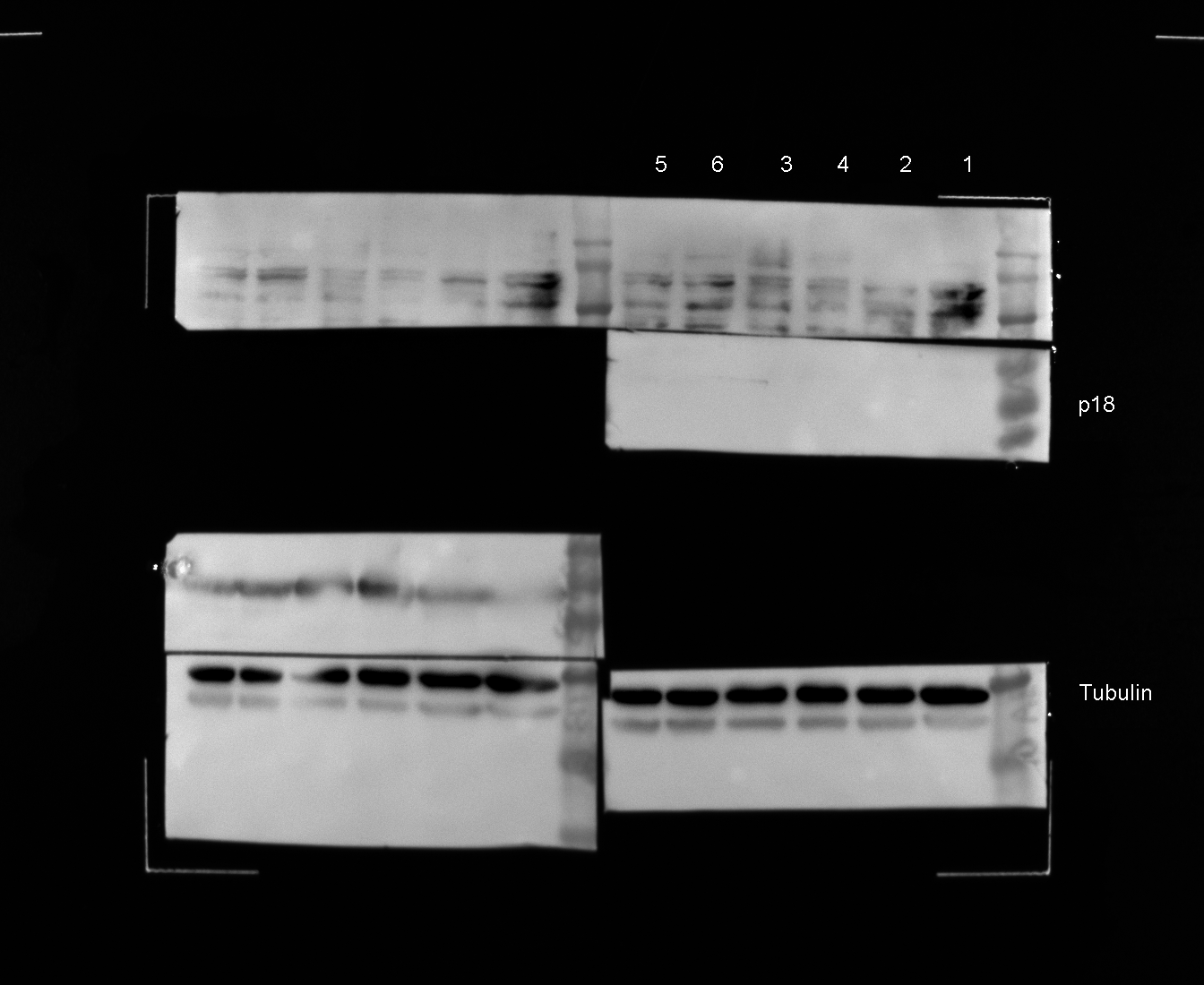

Supplement: Supplemental Information 1 [file peerj-10-13535-s001.zip › Original WB pictures/Fig.2F7-P18+Tubulin exposure 1.tif]

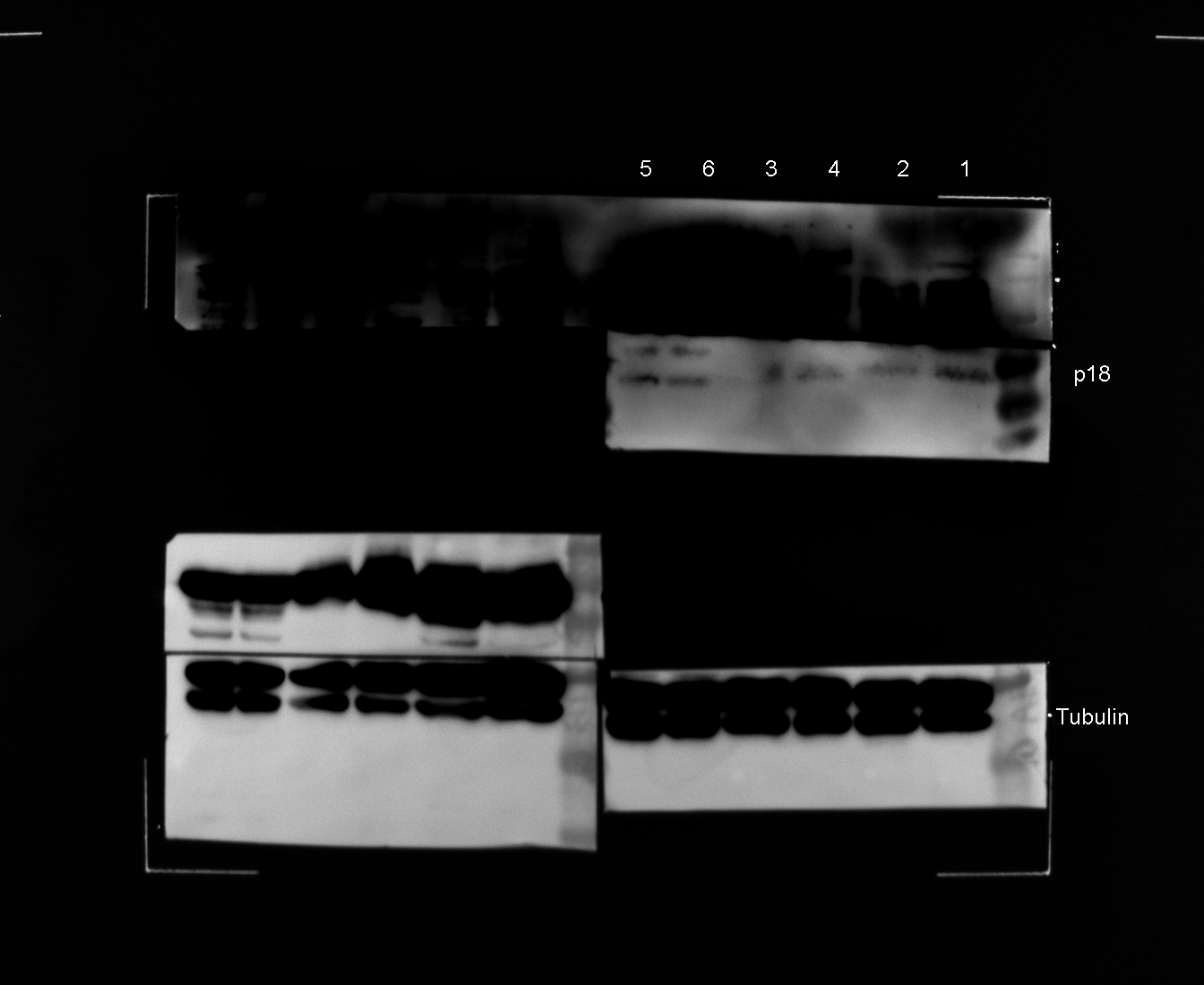

Supplement: Supplemental Information 1 [file peerj-10-13535-s001.zip › Original WB pictures/Fig.2F7-P18+Tubulin exposure 2.tif]

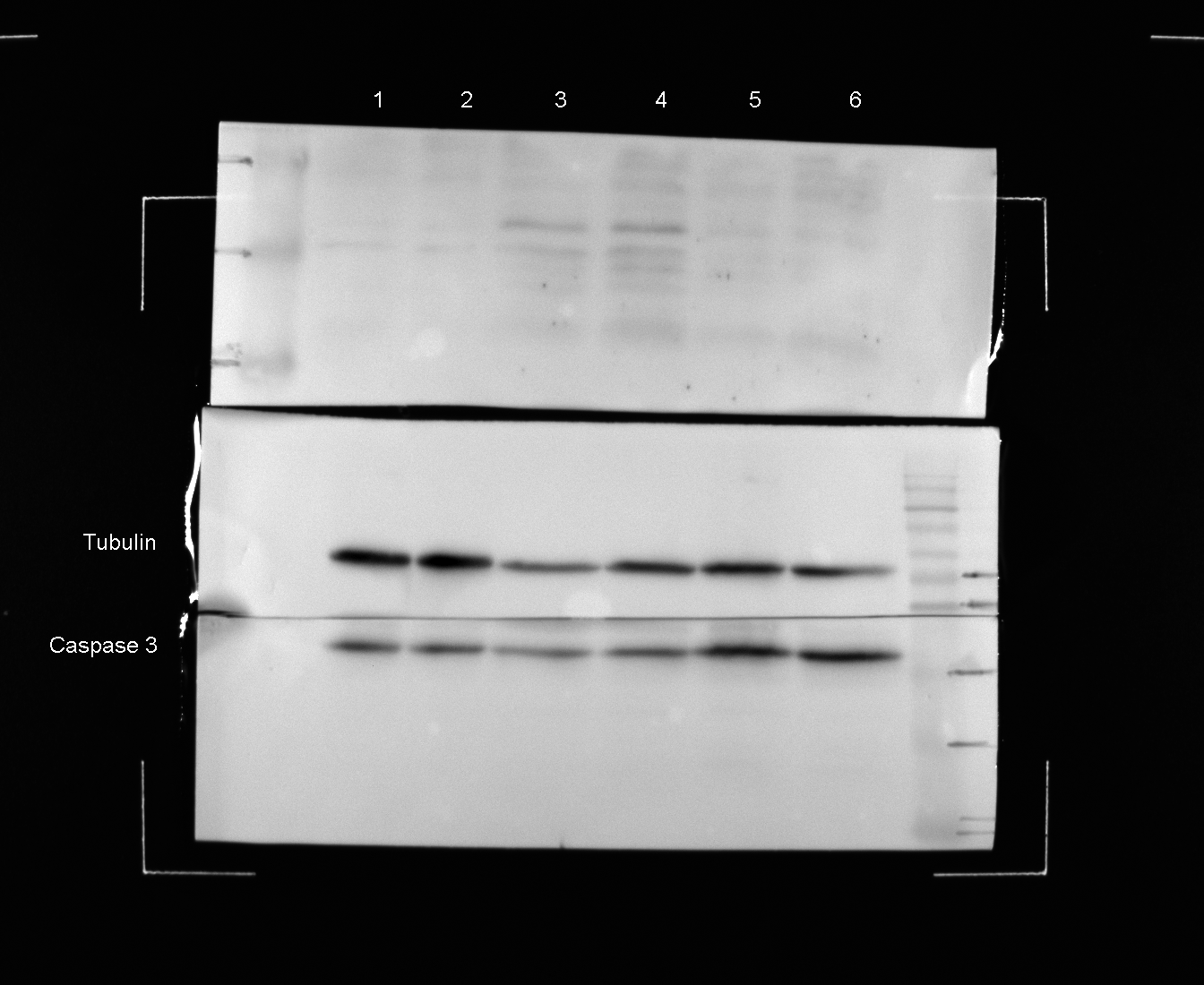

Supplement: Supplemental Information 1 [file peerj-10-13535-s001.zip › Original WB pictures/Fig.3D1-Caspase 3+Tubulin.tif]

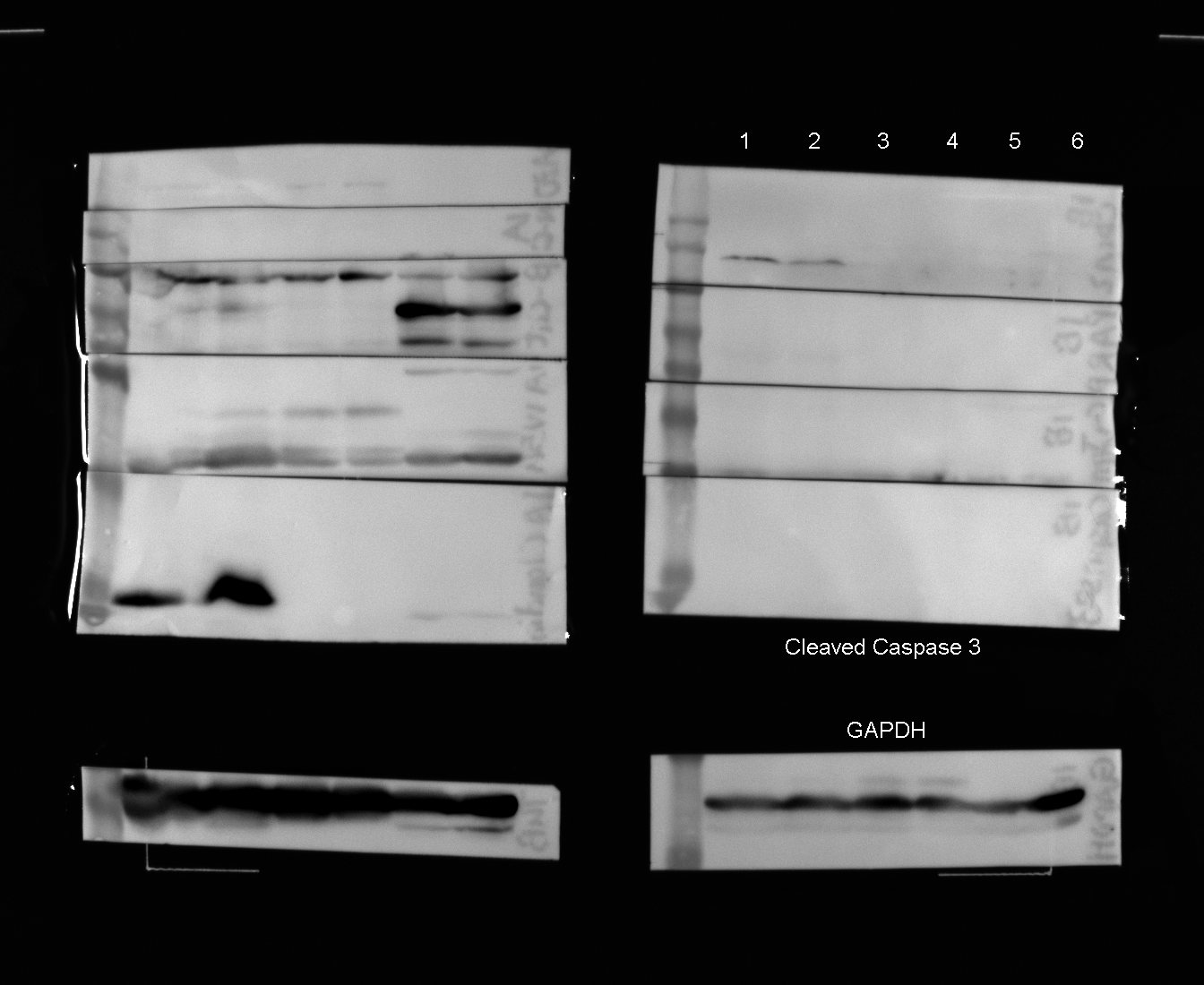

Supplement: Supplemental Information 1 [file peerj-10-13535-s001.zip › Original WB pictures/Fig.3D2-Cleaved caspase3+GAPDH exposure 1.tif]

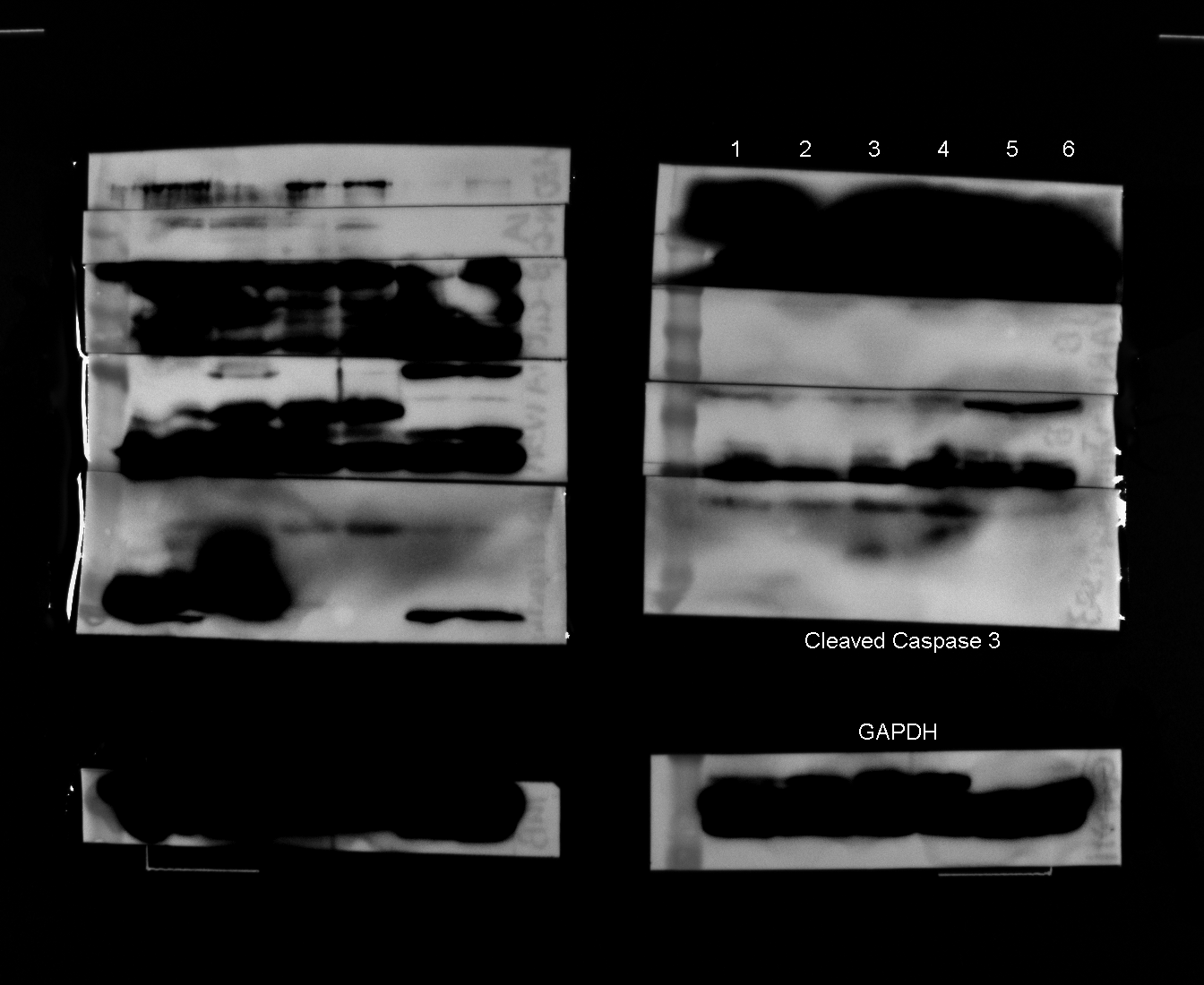

Supplement: Supplemental Information 1 [file peerj-10-13535-s001.zip › Original WB pictures/Fig.3D2-Cleaved caspase3+GAPDH exposure 2.tif]

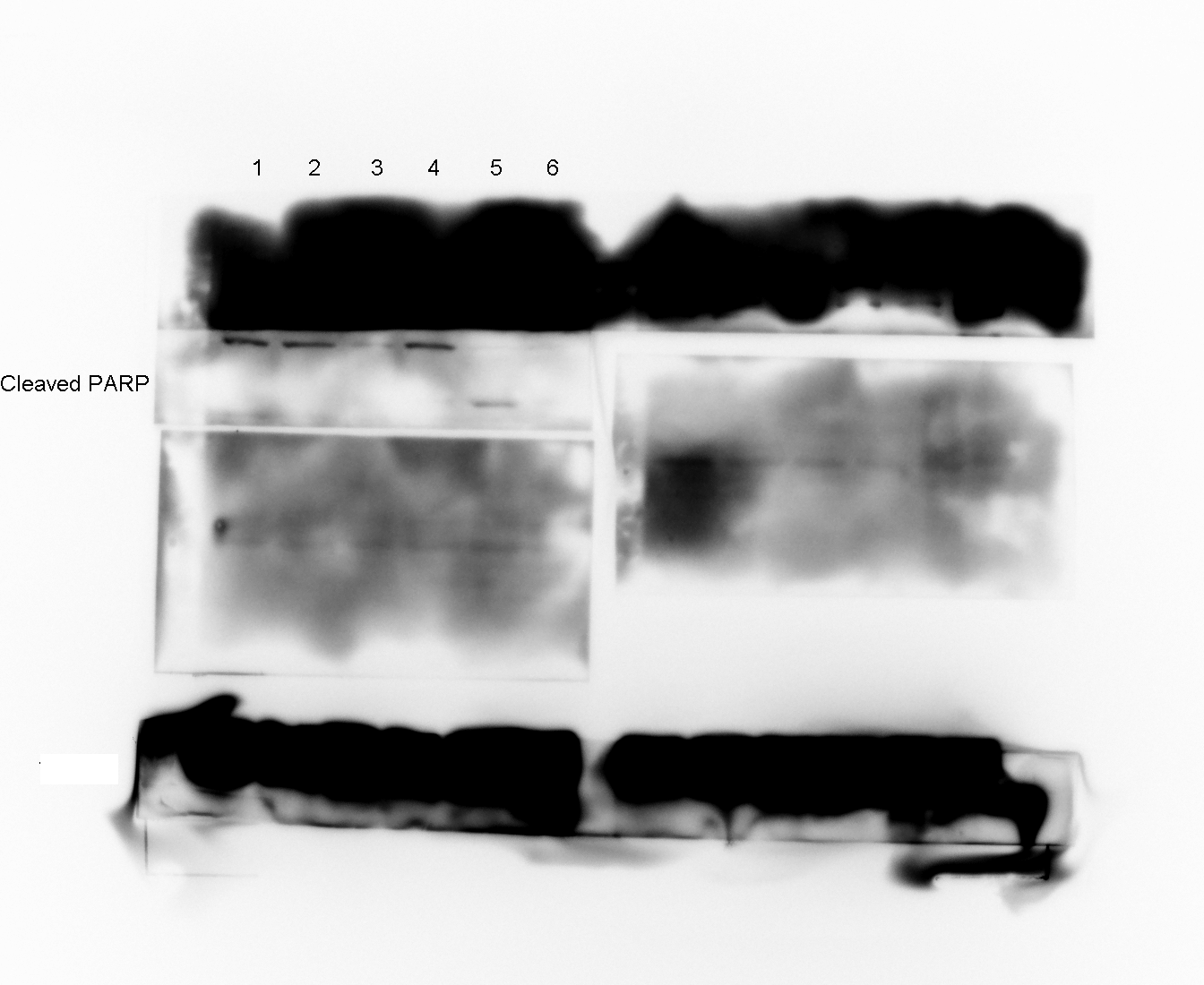

Supplement: Supplemental Information 1 [file peerj-10-13535-s001.zip › Original WB pictures/Fig.3D3-Cleaved-PARP.tif]

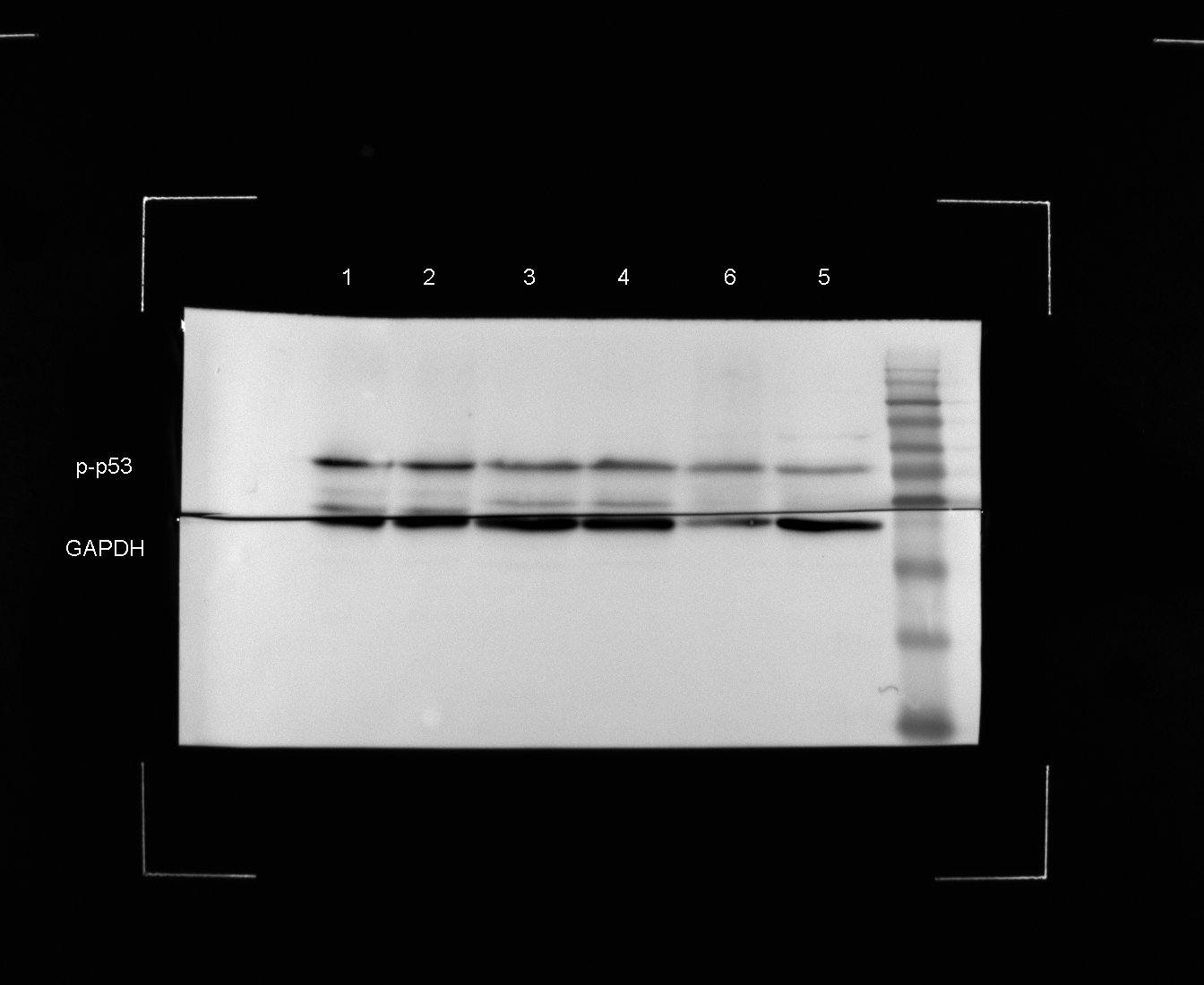

Supplement: Supplemental Information 1 [file peerj-10-13535-s001.zip › Original WB pictures/Fig.3D4-p-p53+GAPDH.tif]

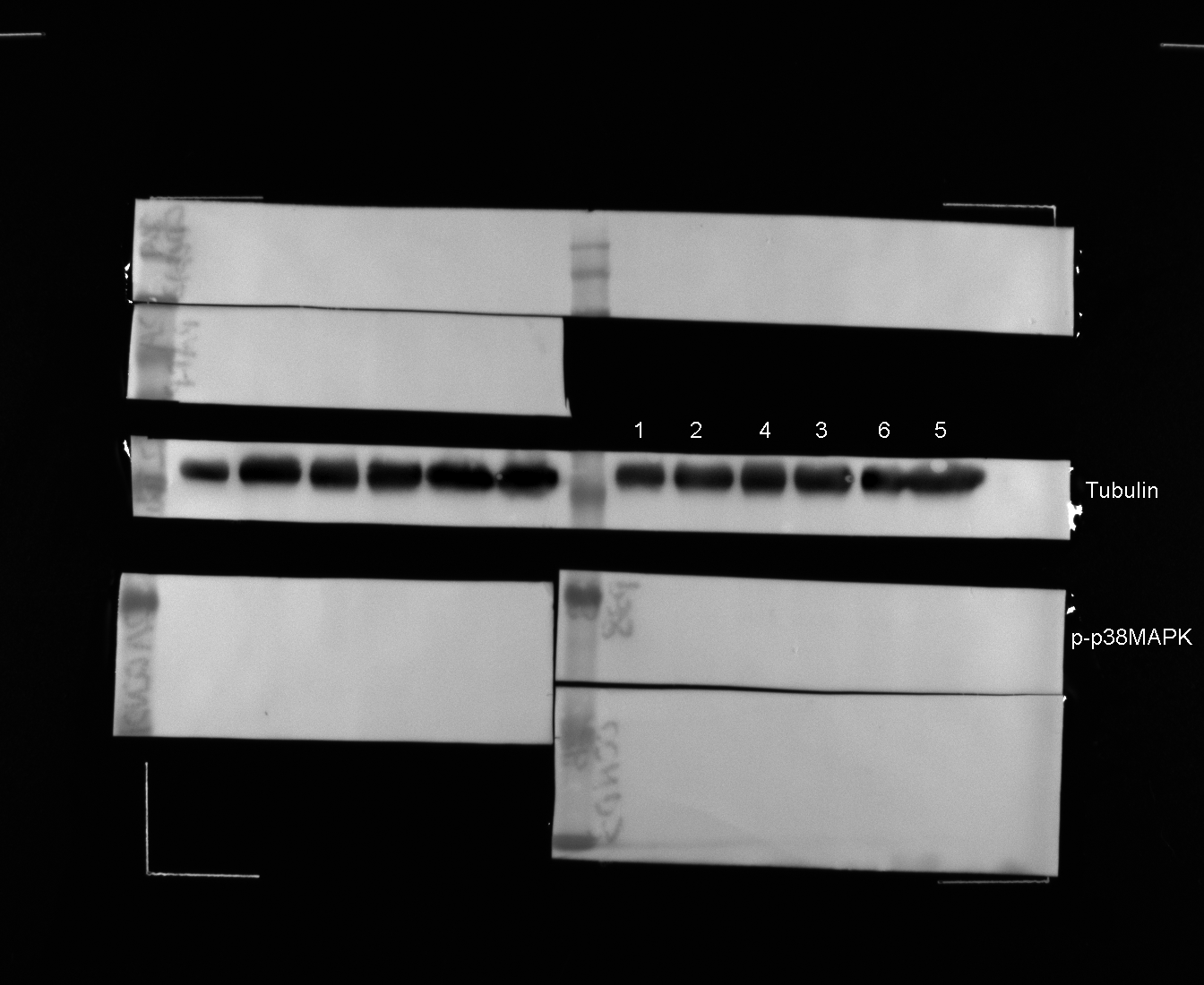

Supplement: Supplemental Information 1 [file peerj-10-13535-s001.zip › Original WB pictures/Fig.3D5-p-p38MAPK exposure 1.tif]

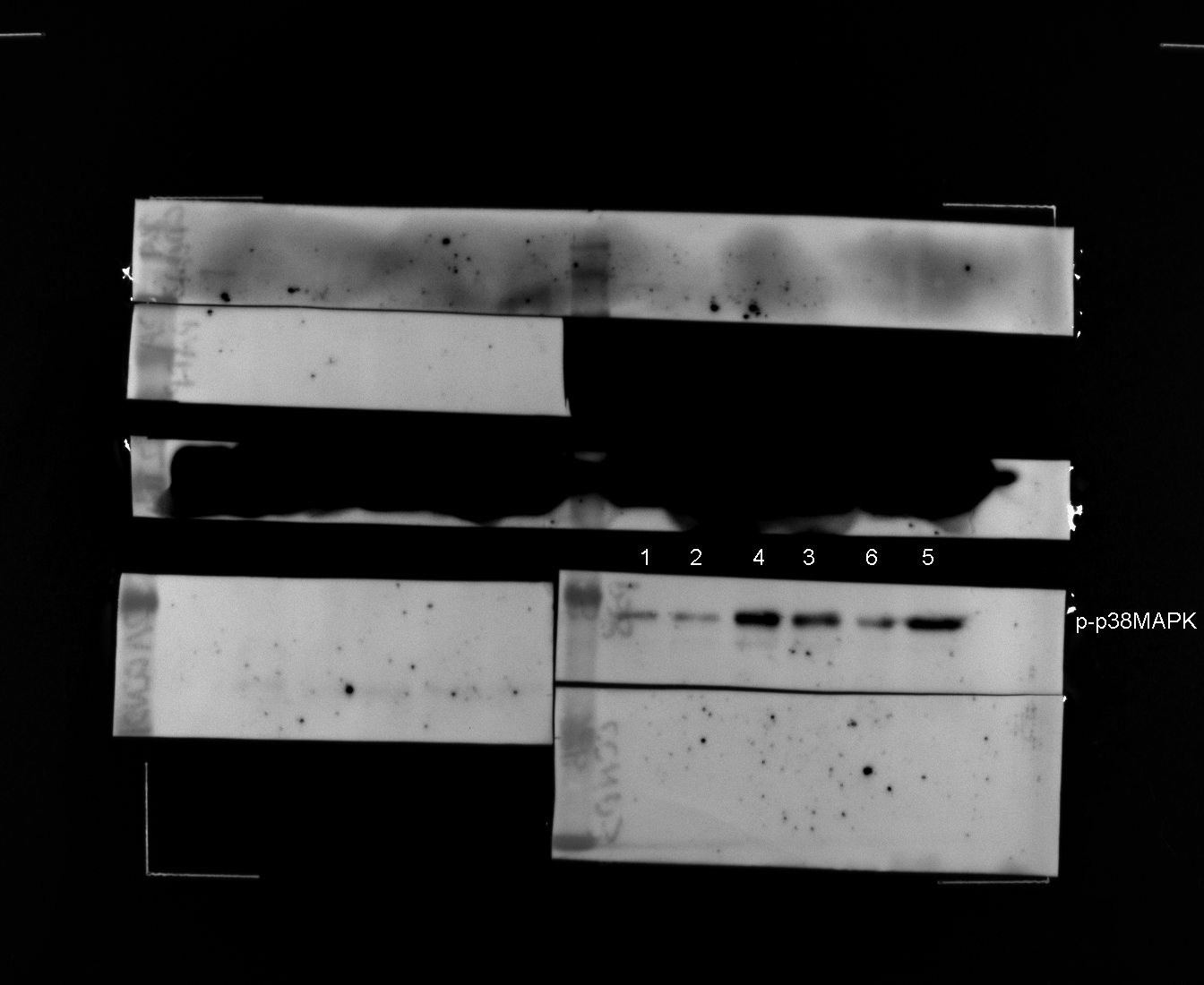

Supplement: Supplemental Information 1 [file peerj-10-13535-s001.zip › Original WB pictures/Fig.3D5-p-p38MAPK exposure 2.tif]

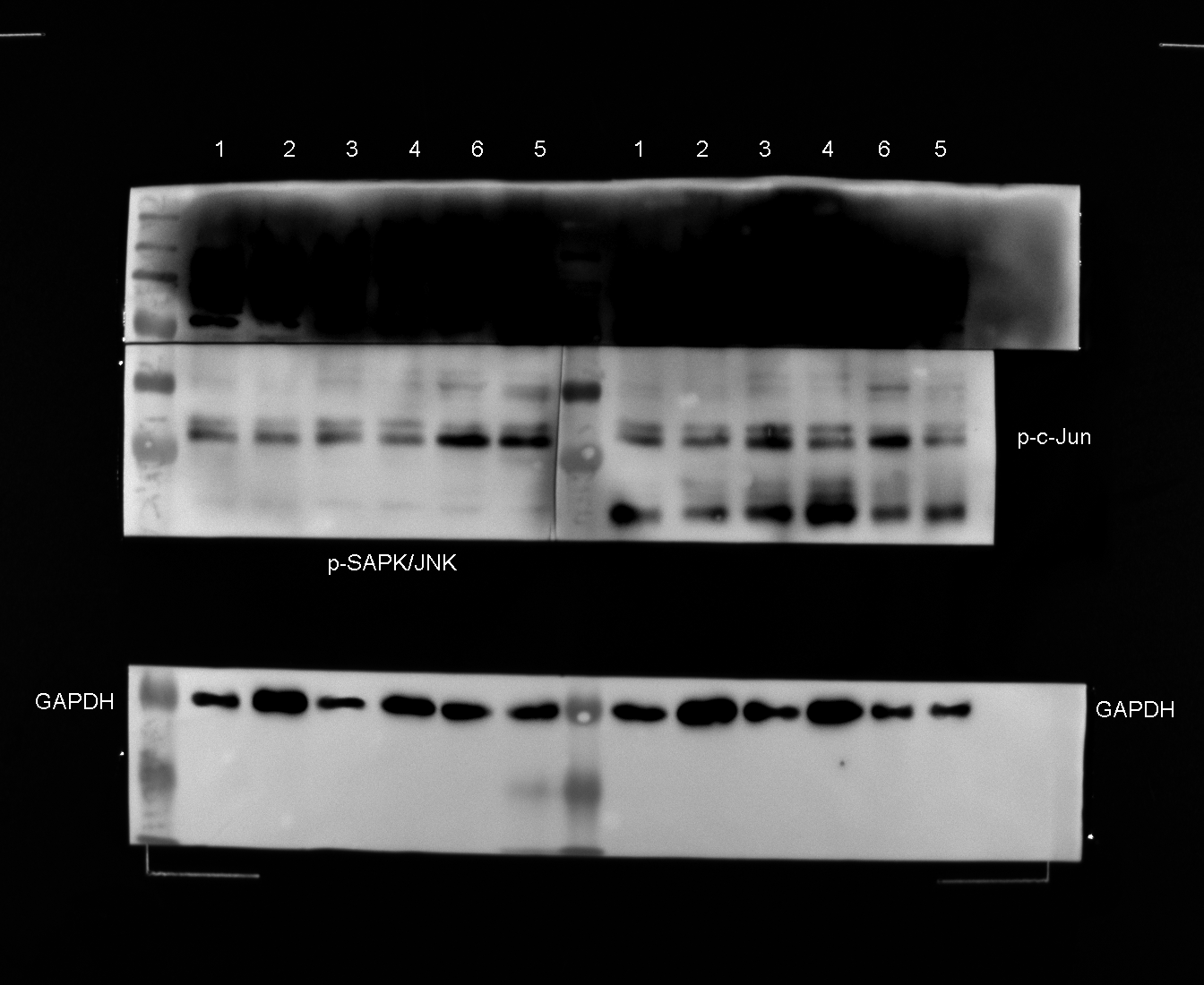

Supplement: Supplemental Information 1 [file peerj-10-13535-s001.zip › Original WB pictures/Fig.3D6-p-SAPKJNK+p-c-Jun+GAPDH.tif]

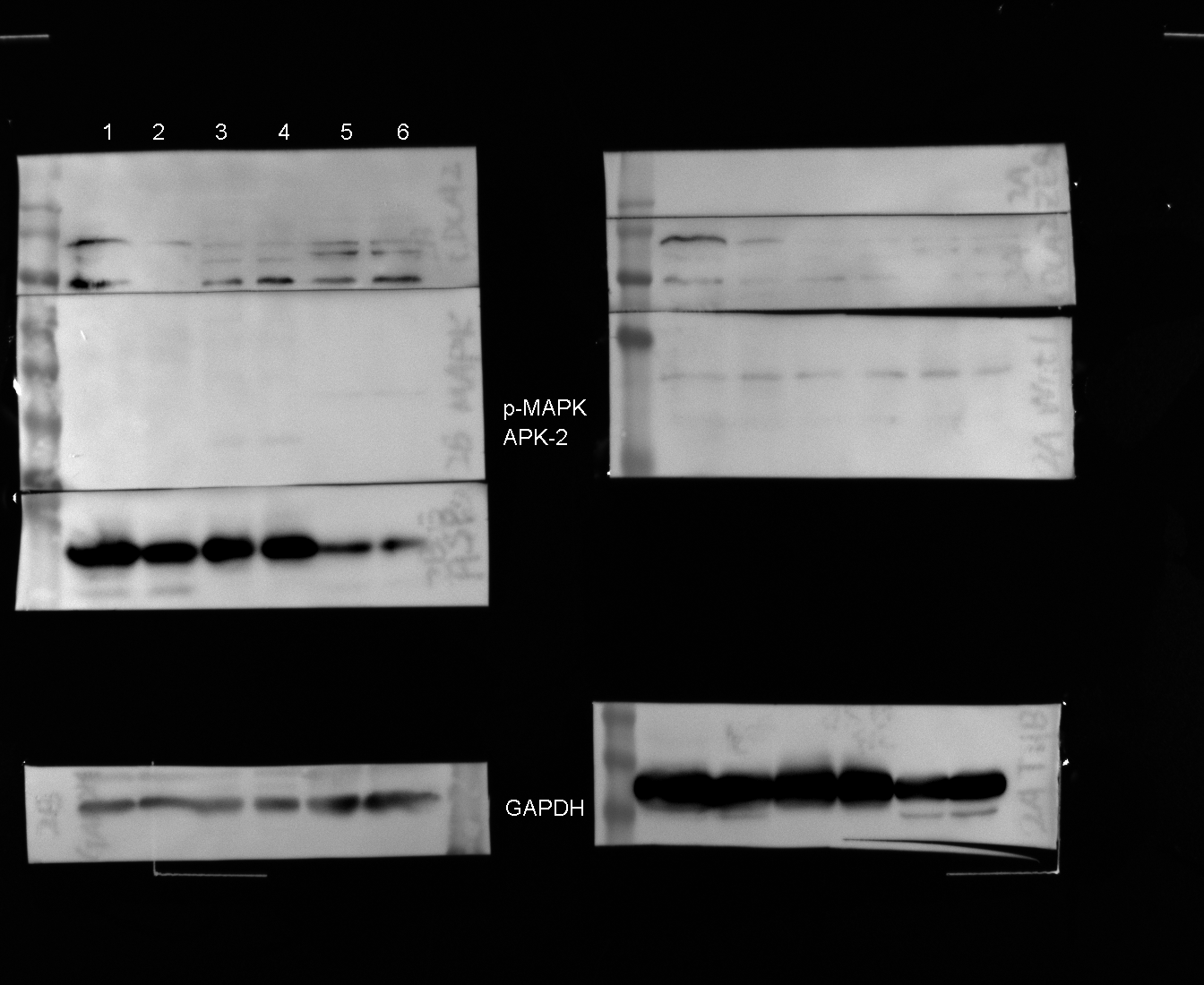

Supplement: Supplemental Information 1 [file peerj-10-13535-s001.zip › Original WB pictures/Fig.3D7-p-MAPKAPK-2+p-HSP27+GAPDH exposure 1.tif]

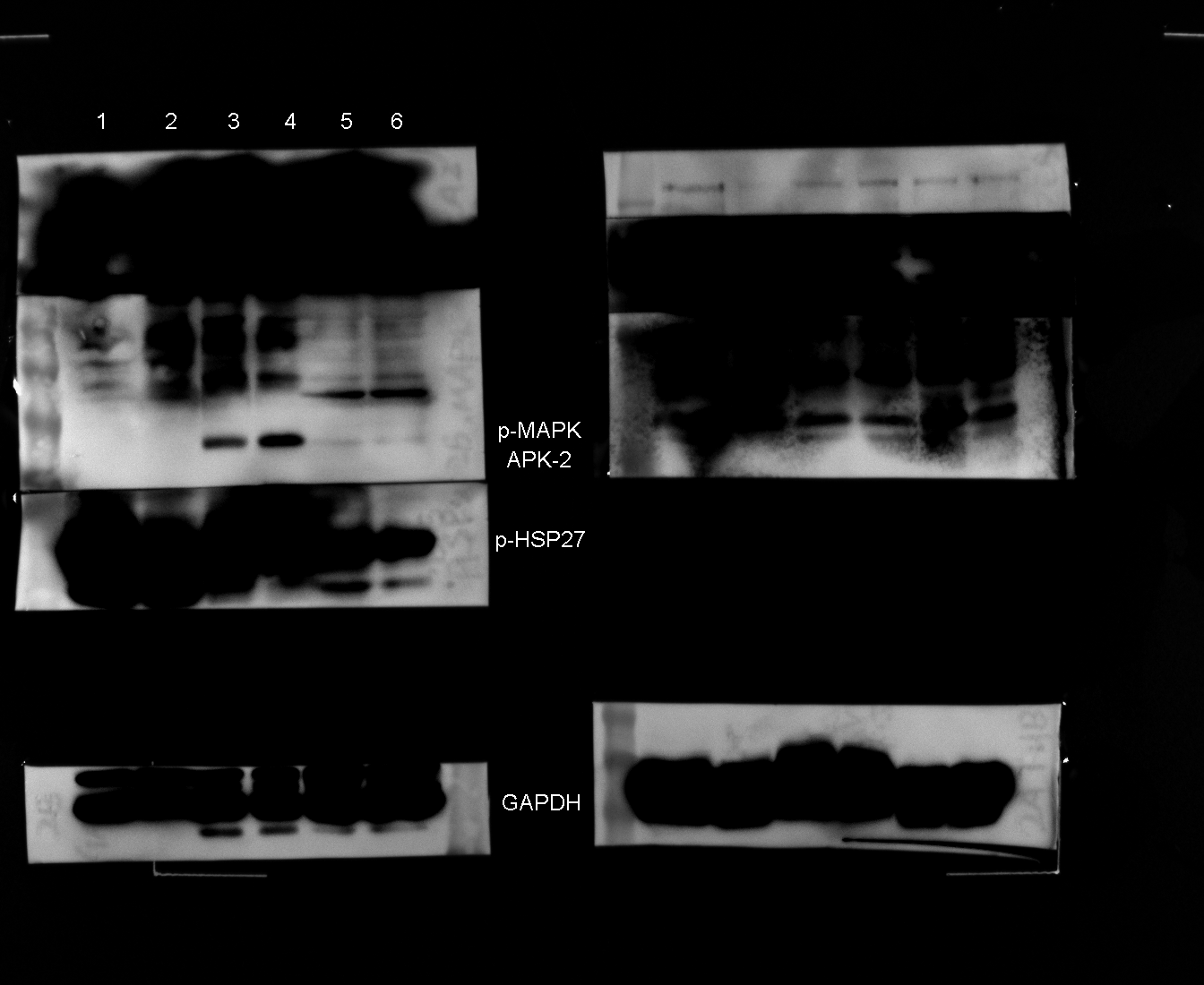

Supplement: Supplemental Information 1 [file peerj-10-13535-s001.zip › Original WB pictures/Fig.3D7-p-MAPKAPK-2+p-HSP27+GAPDH exposure 2.tif]

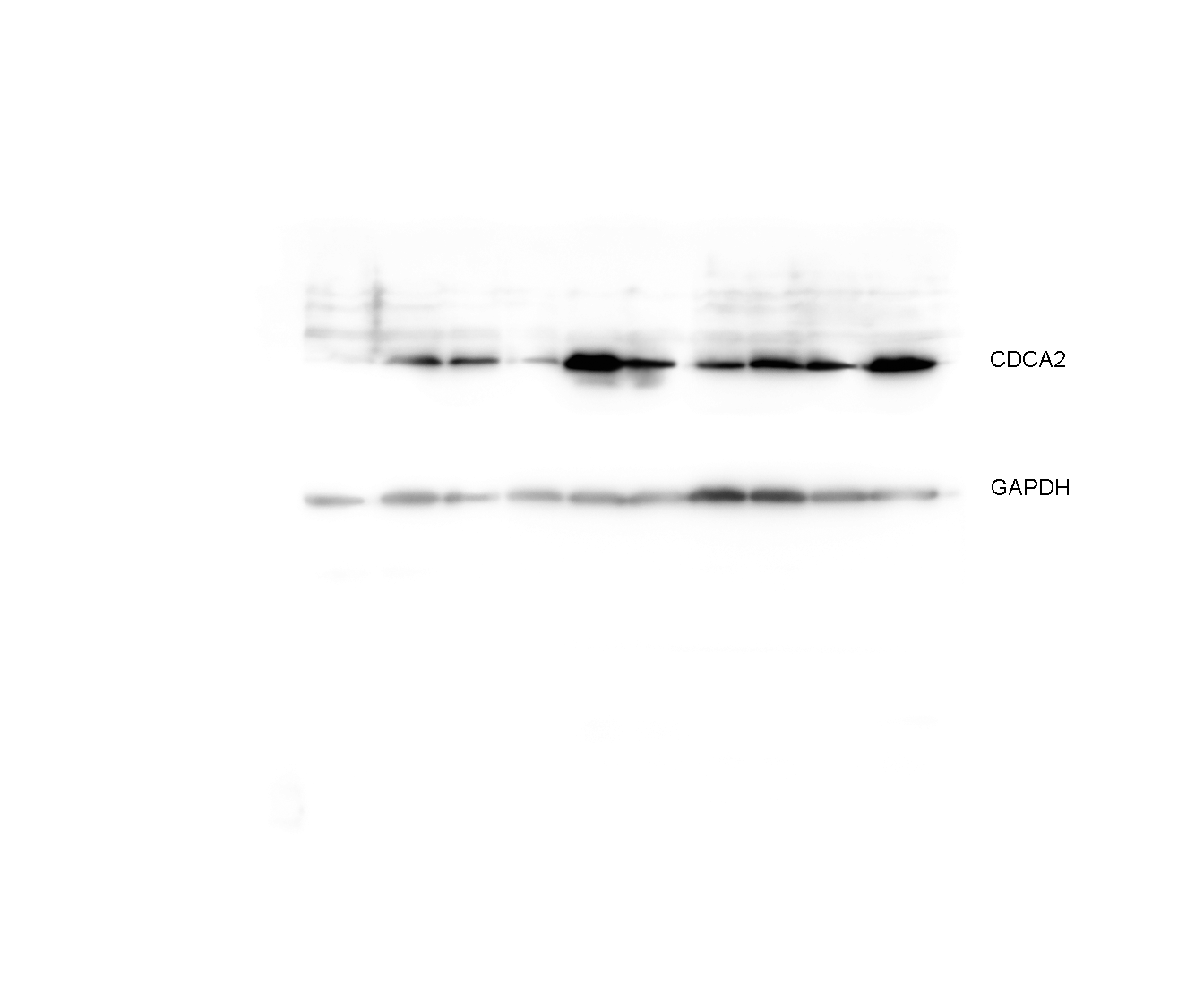

Supplement: Supplemental Information 1 [file peerj-10-13535-s001.zip › Original WB pictures/Fig.4D1-CDCA2+GAPDH.tif]

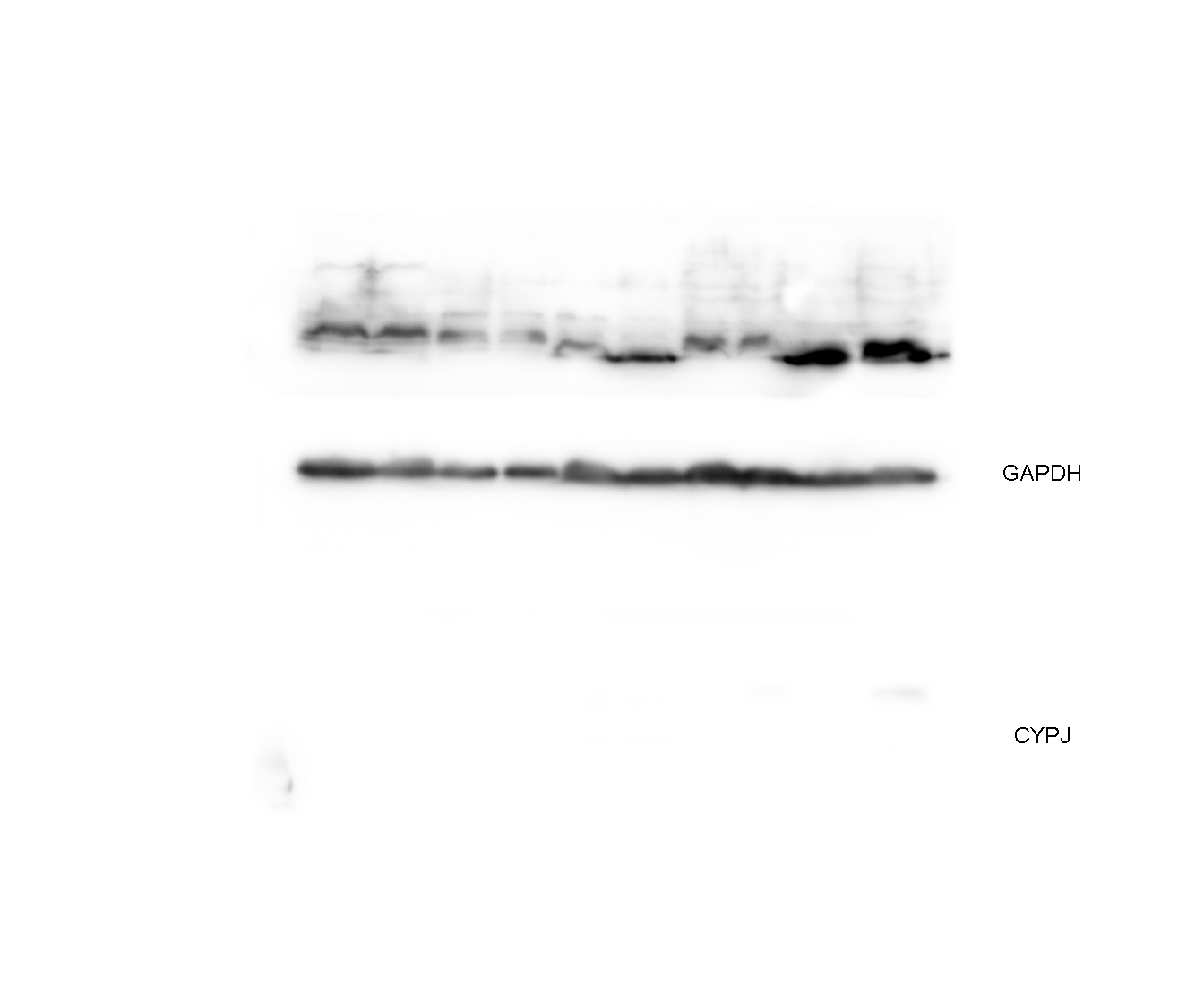

Supplement: Supplemental Information 1 [file peerj-10-13535-s001.zip › Original WB pictures/Fig.4D2-CYPJ+GAPDH exposure 1.tif]

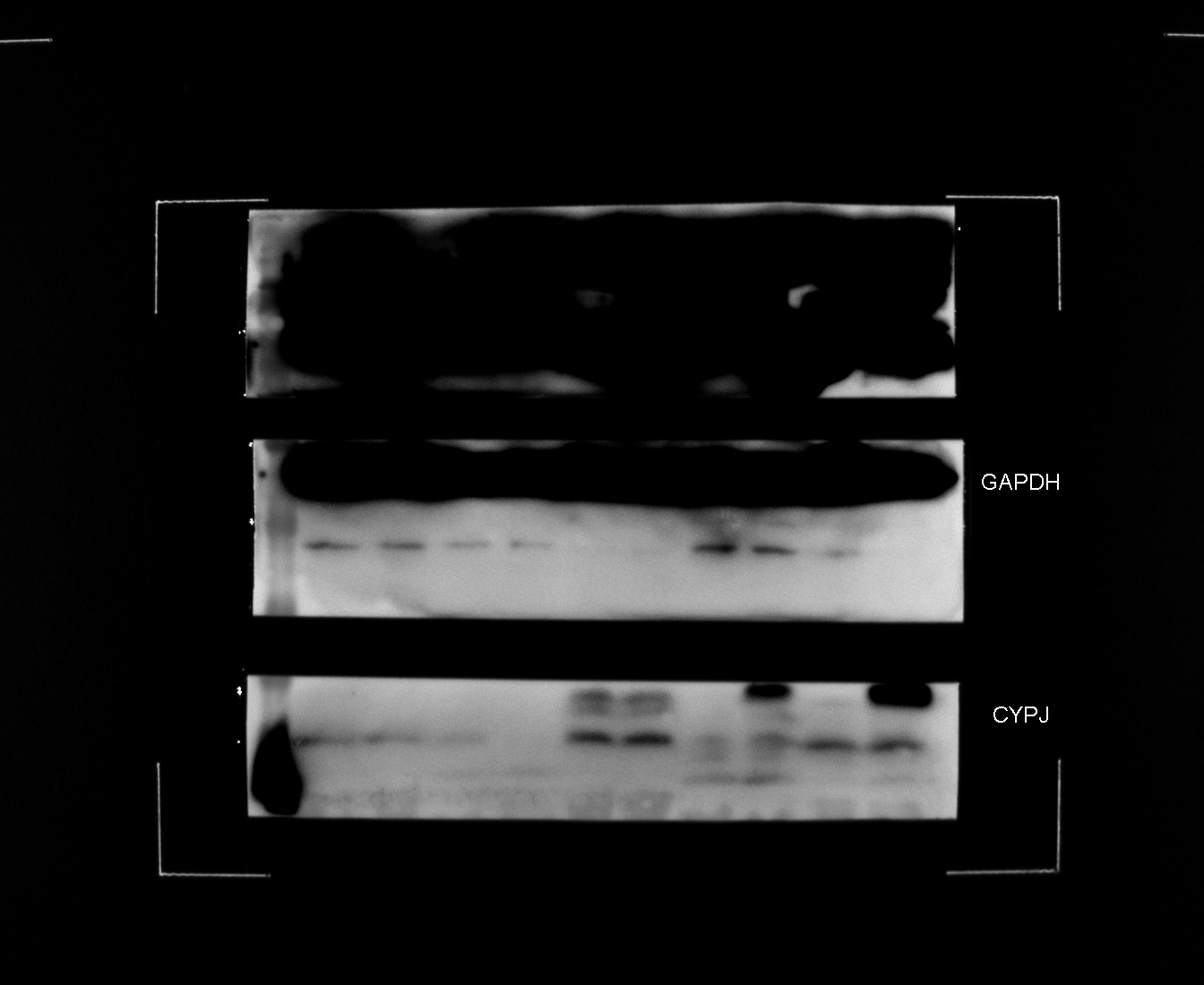

Supplement: Supplemental Information 1 [file peerj-10-13535-s001.zip › Original WB pictures/Fig.4D2-CYPJ+GAPDH exposure 2.tif]
